# Influence of duck eggshell powder modifications by the calcination process or addition of iron (III) oxide-hydroxide on lead removal efficiency

**DOI:** 10.1038/s41598-023-39325-w

**Published:** 2023-07-26

**Authors:** Pornsawai Praipipat, Pimploy Ngamsurach, Rattanaporn Tannadee

**Affiliations:** 1 Department of Environmental Science, Faculty of Science, Khon Kaen University, Khon Kaen, 40002 Thailand; 2 Environmental Applications of Recycled and Natural Materials (EARN) Laboratory, Khon Kaen University, Khon Kaen, 40002 Thailand

**Keywords:** Engineering, Materials science

## Abstract

Lead-contaminated wastewater causes toxicity to aquatic life and water quality for water consumption, so it is required to treat wastewater to be below the water quality standard before releasing it into the environment. Duck eggshell powder (DP), duck eggshell powder mixed iron (III) oxide-hydroxide (DPF), calcinated duck eggshell powder (CDP), and calcinated duck eggshell powder mixed iron (III) oxide-hydroxide (CDPF) were synthesized, characterized, and investigated lead removal efficiencies by batch experiments, adsorption isotherms, kinetics, and desorption experiments. CDPF demonstrated the highest specific surface area and pore volume with the smallest pore size than other materials, and they were classified as mesoporous materials. DP and DPF demonstrated semi-crystalline structures with specific calcium carbonate peaks, whereas CDP and CDPF illustrated semi-crystalline structures with specific calcium oxide peaks. In addition, the specific iron (III) oxide-hydroxide peaks were detected in only DPF and CDPF. Their surface structures were rough with irregular shapes. All materials found carbon, oxygen, and calcium, whereas iron, sodium, and chloride were only found in DPF and CDPF. All materials were detected O–H, C=O, and C–O, and DPF and CDPF were also found Fe–O from adding iron (III) oxide-hydroxide. The point of zero charges of DP, DPF, CDP, and CDPF were 4.58, 5.31, 5.96, and 6.75. They could adsorb lead by more than 98%, and CDPF illustrated the highest lead removal efficiency. DP and CDP corresponded to the Langmuir model while DPF and CDPF corresponded to the Freundlich model. All materials corresponded to a pseudo-second-order kinetic model. Moreover, they could be reusable for more than 5 cycles for lead adsorption of more than 73%. Therefore, CDPF was a potential material to apply for lead removal in industrial applications.

## Introduction

The release of lead-contaminated wastewater from battery, steel, dye and pigment, plastic, and electronic industries causes environmental problems through its toxicity to aquatic life and water quality to water consumption. In addition, the dysfunctional systems of nerves, reproductive, respiration, blood, and many diseases of anemia, lead poisoning, and Alzheimer have been caused by receiving lead into the human body^1^. Therefore, it recommends removing lead from wastewater under the water quality standard which does not exceed 0.2 mg/L following USEPA standards before releasing it into the environment.

Many methods have been applied for eliminating heavy metals in wastewater such as chemical precipitation, oxidation–reduction, coagulation-flocculation, and ion exchange; however, they also leave many concerns of incomplete treatment, expensive operating costs, and creating toxic sludges^2^. As a result, an alternative method of adsorption method is a good choice because it is an efficient and simple method with suitable operating cost including many choices of adsorbents to deal with the specific target pollutants. Various food wastes to eliminate heavy metals in wastewater in 2020–2022 are illustrated in Table 1. In the case of lead removals, eggshells are popularly used because they consist of calcium carbonate (CaCO_3_) and a hydroxyl group (–OH) which could highly adsorb lead in wastewater. Especially, duck eggshells are more CaCO_3_ content and porous than chicken eggshells^3,4^, so they could possibly remove higher lead than chicken eggshells. Moreover, no study has yet used duck eggshells for lead removal in wastewater. As a result, duck eggshells are a good alternative adsorbent among those adsorbents mentioned in Table 1. However, duck eggshells also need to improve efficiency to deal with a high lead strength concentration in industrial wastewater.

**Table 1:** Various food wastes for eliminating heavy metals in wastewater.

Materials	Heavy metals	*q*_m_ (mg/g)	References
Papaya peel	Lead (Pb^2+^)	–	^5^
Lemon peel	Lead (Pb^2+^)	–	^6^
*Citrus limon* peel	Lead (Pb^2+^)	100	^7^
*Citrus limon* peel	Copper (Cu^2+^)	76.92	^7^
*Citrus limon* peel	Chromium (Cr^3+^)	111.11	^7^
Orange peel	Lead (Pb^2+^)	67.15	^8^
Orange peel	Copper (Cu^2+^)	54.94	^8^
Banana peel	Lead (Pb^2+^)	39.32	^9^
Banana peel	Copper (Cu^2+^)	29.26	^9^
Pomegranate peel	Copper (Cu^2+^)	–	^10^
Pomegranate peel	Cadmium (Cd^2+^)	–	^10^
Pomegranate peel	Zinc (Zn^2+^)	–	^10^
Melon peel	Lead (Pb^2+^)	191.93	^11^
Melon peel	Copper (Cu^2+^)	77.76	^11^
Melon peel	Cadmium (Cd^2+^)	76.16	^11^
Pistachio shell	Cadmium (Cd^2+^)	51.28	^12^
Peanut shell	Cadmium (Cd^2+^)	62.11	^12^
Almond shell	Cadmium (Cd^2+^)	78.74	^12^
Chicken eggshell	Lead (Pb^2+^)	18.80	^13^
Chicken eggshell	Lead (Pb^2+^)	25.19	^14^
Chicken eggshell	Lead (Pb^2+^)	180.5	^15^
Chicken eggshell	Lead (Pb^2+^)	277.78	^16^
Chicken eggshell	Cadmium (Cd^2+^)	13.62	^16^

Many modification methods which are pyrolysis, calcination, acid or alkaline treatment, and metal oxides have been used to improve material efficiencies of food wastes for heavy metal removals reported in Table 2. Among those metals, both the calcination process and adding metal oxides have been popularly used for increasing the adsorption capacity of heavy metal adsorbents. Thus, it is an interesting point to improve duck eggshell efficiency by using a calcination process or adding iron (III) oxide-hydroxide to confirm whether these two methods increase lead removal efficiency. In addition, no one to modify duck eggshell material with a calcination process along with adding iron (III) oxide-hydroxide. Therefore, this study is the first effort to synthesize duck eggshell materials with or without a calcination process or adding iron (III) oxide-hydroxide, to compare their lead removal efficiencies through batch experiments, and verify whether using a calcination process or the addition of iron (III) oxide-hydroxide increases material adsorption capacity.

**Table 2:** The modification methods for improving material efficiencies of food wastes for heavy metal removals.

Modifications	Materials	Metal metals	*q*_m_ (mg/g)	References
Biochar	Eggshell	Lead (Pb^2+^)	16.41–104.25	^17^
	Banana peel	Lead (Pb^2+^)	315.16	^18^
	Pomelo peel	Lead (Pb^2+^)	102.39	^19^
	Pomelo peel	Copper (Cu^2+^)	47.54	^19^
Calcination	Eggshell	Lead (Pb^2+^)	33.90	^20^
	Chicken eggshell	Lead (Pb^2+^)	29.60	^21^
	Chicken eggshell	Cadmium (Cd^2+^)	66.01	^21^
Potassium permanganate (KMnO_4_)	Eggshell	Lead (Pb^2+^)	709.13	^22^
Hydrochloric acid (HCl)	Eggshell	Lead (Pb^2+^)	16.95	^23^
Sodium hydroxide (NaOH)	Banana peel	Lead (Pb^2+^)	89.29	^24^
	Banana peel	Copper (Cu^2+^)	5.72	^24^
	*Citrullus lanatus* peel	Cadmium (Cd^2+^)	19.31	^25^
	*Citrullus lanatus* peel	Lead (Pb^2+^)	25.78	^25^
	Coconut coir	Copper (Cu^2+^)	–	^26^
Aluminum chloride (AlCl_3_) and Manganese(II) chloride (MnCl_2_)	Corncob	Cadmium (Cd^2+^)	45.58	^27^
Iron(III) oxide-hydroxide	Lemon peel	Lead (Pb^2+^)	3.52	^28^
	Chicken eggshell	Lead (Pb^2+^)	42.74	^14^
Magnetic	Chicken eggshell	Chromium (Cr^6+^)	111.11	^29^
	Eggshell	Lead (Pb^2+^)	0.07	^30^
Iron(II,III) oxide (Fe_3_O_4_)	Eggshell	Cadmium (Cd^2+^)	–	^31^
	Eggshell	Lead (Pb^2+^)	57.14	^32^
	Peanut shell	Lead (Pb^2+^)	188.68	^33^

Duck eggshell powder (DP), duck eggshell powder mixed iron (III) oxide-hydroxide (DPF), calcined duck eggshell powder (CDP), and calcined duck eggshell powder mixed iron (III) oxide-hydroxide (CDPF) were synthesized and characterize their specific surface area, pore volumes, pore sizes, crystalline formations, surface morphologies, chemical elements, and chemical functional groups by Brunauer–Emmett–Teller (BET), X-ray diffractometer (XRD), Field emission scanning electron microscopy and focus ion beam (FESEM-FIB) with energy dispersive X-ray spectrometer (EDX), and Fourier transform infrared spectroscopy (FT-IR). The point of zero charges and lead removal efficiencies of DP, DPF, CDP, and CDPF by batch experiments with varying doses, contact time, pH, and concentration were investigated. In addition, linear and nonlinear adsorption isotherms of Langmuir, Freundlich, Temkin, and Dubinin-Radushkevich models were used to determine their lead adsorption patterns. Moreover, linear and nonlinear pseudo-first-kinetic, pseudo-second-kinetic, elovich, and intraparticle diffusion models were used to identify their rates and mechanisms for lead adsorptions. Finally, the desorption experiments were used to confirm material reusability.

## Materials and methods

### Raw material

Duck eggshells used in this study are wastes from the local restaurants in Khon Kaen province, Thailand.

### Chemicals

Ferric chloride hexahydrate (FeCl_3_·6H_2_O) (LOBA, India), sodium hydroxide (NaOH) (RCI Labscan, Thailand), sodium chloride (NaCl) (RCI Labscan, Thailand), 37% hydrochloric acid (HCl) (RCI Labscan, Thailand), 65% nitric acid (HNO_3_) (Merck, Germany), and lead nitrate (Pb(NO_3_)_2_) (QRëC, New Zealand) were used, and they were analytical grades (AR) without purification before use. 1% NaOH and 1% HNO_3_ were used for pH adjustments.

### Synthesis of duck eggshell materials

**Figure 1:**
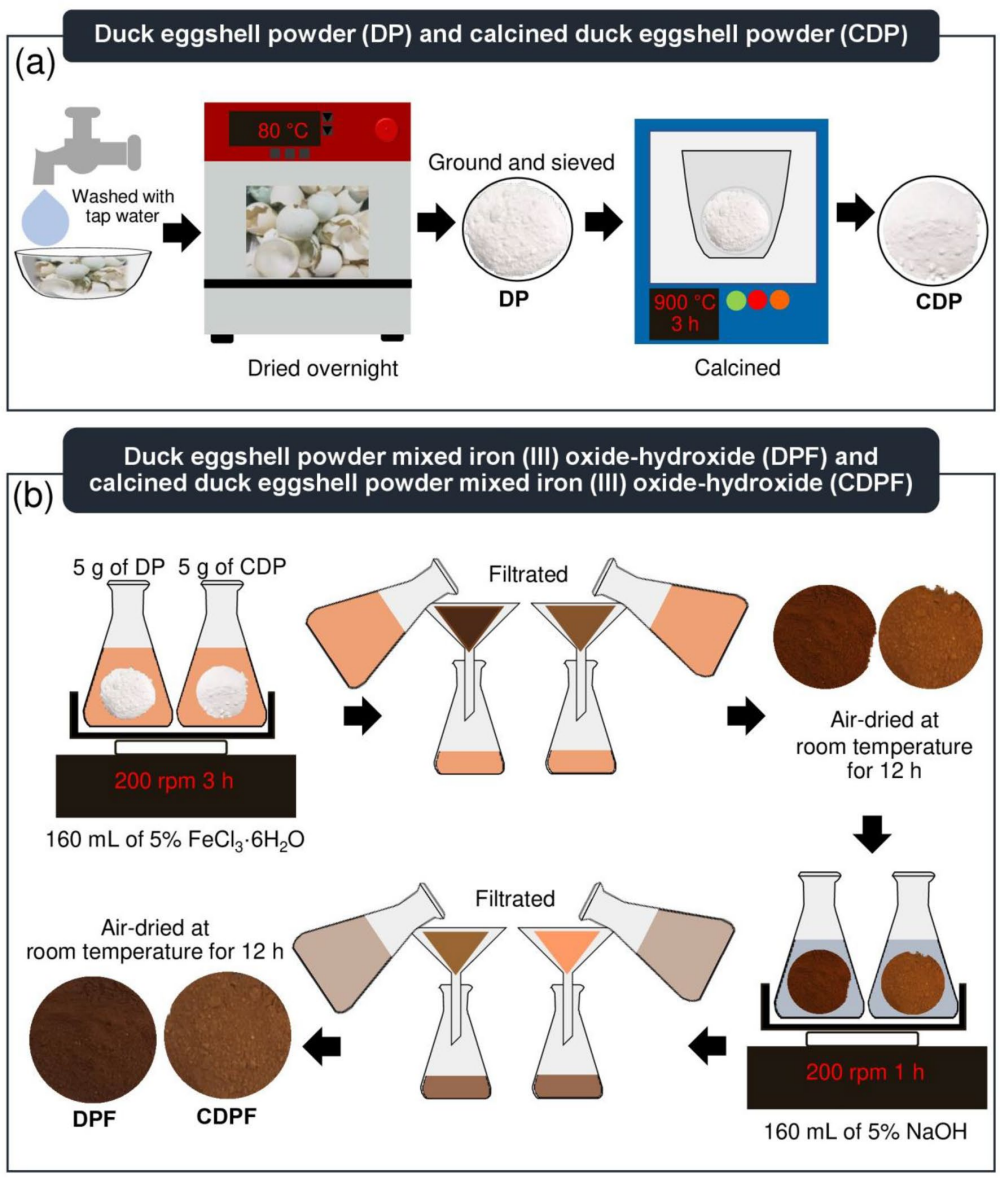
The synthesis methods of (**a**) duck eggshell powder (DP) and calcined duck eggshell powder (CDP) (**b**) duck eggshell powder mixed iron (III) oxide-hydroxide (DPF) and calcined duck eggshell powder mixed iron (III) oxide-hydroxide (CDPF).

The synthesis methods of duck eggshell materials of duck eggshell powder (DP), duck eggshell powder mixed iron (III) oxide-hydroxide (DPF), calcined duck eggshell powder (CDP), and calcined duck eggshell powder mixed iron (III) oxide-hydroxide (CDPF) are illustrated in Fig. 1a,b which is based on Praipipat et al. (2022)^34^ and Praipipat et al. (2023)^14^, and the details were described below:

#### The synthesis of duck eggshell powder (DP) and calcined duck eggshell powder (CDP)

For DP, duck eggshells were washed with tap water to eliminate contaminations, and then they were dried overnight in a hot air oven (Binder, FED 53, Germany) at 80 °C. Next, they were ground and sieved in size of 125 µm. Then, they were kept in a desiccator before use called duck eggshell powder (DP). For CDP, it was calcined by a furnace (Chavachote, L9/12P, Thailand) in an air atmosphere at 900 °C for 3 h, and then they were kept in a desiccator before use called calcined duck eggshell powder (CDP).

#### The synthesis of duck eggshell powder mixed iron (III) oxide-hydroxide (DPF) and calcined duck eggshell powder mixed iron (III) oxide-hydroxide (CDPF)

Firstly, 5 g of DP or CDP were added to 500 mL of Erlenmeyer flask containing 160 mL of 5% FeCl_3_·6H_2_O, and they were mixed by an orbital shaker (GFL, 3020, Germany) of 200 rpm for 3 h. Next, they were filtrated and air-dried at room temperature for 12 h. Then, they were added to 500 mL of Erlenmeyer flask containing 160 mL of 5% NaOH, and they were mixed by an orbital shaker of 200 rpm for 1 h. After that, they were filtered and air-dried at room temperature for 12 h. Finally, they were kept in a desiccator before use called duck eggshell powder mixed iron (III) oxide-hydroxide (DPF) or calcined duck eggshell powder mixed iron (III) oxide-hydroxide (CDPF).

### Characterizations of duck eggshell materials

Various characterized techniques were used for characterizing duck eggshell powder (DP), duck eggshell powder mixed iron (III) oxide-hydroxide (DPF), calcined duck eggshell powder (CDP), and calcined duck eggshell powder mixed iron (III) oxide-hydroxide (CDPF). Firstly, Brunauer–Emmett–Teller (BET) (Bel, Bel Sorp mini X, Japan) by isothermal nitrogen gas (N_2_) adsorption–desorption at 77.3 K and degas temperature of 80 °C for 6 h was used to identify their specific surface area, pore volumes, and pore sizes. Second, an X-ray diffractometer (XRD) (PANalytical, EMPYREAN, UK) in a range of 2θ = 5–80° was used for investigating their crystalline structures. Third, Field emission scanning electron microscopy and focus ion beam (FESEM-FIB) with energy dispersive X-ray spectrometer (EDX) (FEI, Helios NanoLab G3 CX, USA) which the samples were placed on aluminum stubs with gold-coating for 4 min using a 108 auto Sputter Coater with thickness controller MTM-20 model (Cressington, Ted Pella Inc, USA) by analyzing at 10 kV accelerating voltage was used for studying their surface morphologies and chemical compositions. Finally, Fourier transform infrared spectroscopy (FT-IR) (Bruker, TENSOR27, Hong Kong) in a range of 600–4000 cm^−1^ with a resolution of 4 cm^−1^ and 16 scans over the entire covered range was used for determining their chemical functional groups.

### The point of zero charges of duck eggshell materials

The points of zero charge of duck eggshell powder (DP), duck eggshell powder mixed iron (III) oxide-hydroxide (DPF), calcined duck eggshell powder (CDP), and calcined duck eggshell powder mixed iron (III) oxide-hydroxide (CDPF) for lead adsorptions were investigated by using 0.1 M NaCl solutions with pH values from 2 to 12 which 0.1 M HCl and 0.1 M NaOH were used for pH adjustments which the method is referred from the study of Praipipat et al.^14^. Firstly, 0.1 g of duck eggshell material was added to 250 mL Erlenmeyer flasks containing 50 mL of each 0.1 M NaCl solution. Then, it was shaken by an orbital shaker (GFL, 3020, Germany) at room temperature at 150 rpm for 24 h. After that, the final pH value of the sample solution was measured by a pH meter (Mettler Toledo, SevenGo with InLab 413/IP67, Switzerland) and ∆pH (pH_final_ – pH_initial_) was calculated. A point that is the crosses line of ∆pH versus pH_initial_ equal to zero is the value of the point of zero charges (pH_pzc_).

### Batch adsorption experiments

Lead removal efficiencies of duck eggshell powder (DP), duck eggshell powder mixed iron (III) oxide-hydroxide (DPF), calcined duck eggshell powder (CDP), and calcined duck eggshell powder mixed iron (III) oxide-hydroxide (CDPF) were investigated through a series of batch experiments with varying values of four affecting factors of adsorbent dosage (2.5–15 g/L), contact time (1–6 h), pH (1, 3, 5, 7, 9), and initial lead concentration (10–70 mg/L). The initial lead concentration of 50 mg/L, a sample volume of 200 mL, pH 5, a shaking speed of 200 rpm, and a temperature of 25 °C were applied as the control condition. The lowest value with the highest lead removal efficiency of each affecting factor was selected as the optimum value, and it was used for the next affecting factor study. The triplicate experiments were conducted for confirming their results. An atomic adsorption spectrophotometer (PerkinElmer, PinAAcle 900 F, USA) was used for analyzing lead concentrations, and Eq. (1) was used to calculate lead removal efficiency in the percentage:

1 $${\text{Lead}}\;{\text{removal}}\;{\text{efficiency}}\;\left( \% \right) = (C_{0} - C_{e} )/C_{0} \times 100$$

where *C*_0_ is the initial lead concentration (mg/L), and *C*_e_ is the equilibrium of lead concentration in the solution (mg/L).

### Adsorption isotherms

The adsorption isotherms of duck eggshell powder (DP), duck eggshell powder mixed iron (III) oxide-hydroxide (DPF), calcined duck eggshell powder (CDP), and calcined duck eggshell powder mixed iron (III) oxide-hydroxide (CDPF) were determined by various adsorption isotherms of linear and nonlinear Langmuir, Freundlich, Temkin, and Dubinin–Radushkevich models to explain their adsorption patterns. In addition, their adsorption isotherms are calculated by Eqs. (2)–(9)^35–38^:

Langmuir isotherm:

2 $${\text{Linear}}:\;C_{{\text{e}}} /q_{{\text{e}}} = 1/q_{{\text{m}}} K_{{\text{L}}} + C_{{\text{e}}} /q_{{\text{m}}}$$

3 $${\text{Nonlinear}}:\;q_{{\text{e}}} = q_{{\text{m}}} K_{{\text{L}}} C_{{\text{e}}} /1 + K_{{\text{L}}} C_{{\text{e}}}$$

Freundlich isotherm:

4 $${\text{Linear}}: \;\log q_{{\text{e}}} = \log K_{{\text{F}}} + 1/n\log C_{{\text{e}}}$$

5 $${\text{Nonlinear}}:\;q_{{\text{e}}} = K_{{\text{F}}} C_{{\text{e}}}^{1/n}$$

Temkin isotherm:

6 $${\text{Linear}}: \;q_{{\text{e}}} = RT/b_{{\text{T}}} \ln A_{{\text{T}}} + RT/b_{{\text{T}}} \ln Ce$$

7 $${\text{Nonlinear}}: \;q_{{\text{e}}} = RT/b_{{\text{T}}} \ln A_{{\text{T}}} C_{{\text{e}}}$$

Dubinin–Radushkevich isotherm:

8 $${\text{Linear}}:\; \ln q_{{\text{e}}} = \ln q_{{\text{m}}} - K_{{{\text{DR}}}} \varepsilon^{2}$$

9 $${\text{Nonlinear}}:\;q_{{\text{e}}} = q_{{\text{m}}} \exp ( - K_{{{\text{DR}}}} \varepsilon^{2} )$$

where *C*_e_ is the equilibrium of lead concentration (mg/L), *q*_e_ is the amount of adsorbed lead on duck eggshell materials (mg/g), *q*_m_ is indicated the maximum amount of lead adsorption on duck eggshell materials (mg/g), *K*_L_ is the adsorption constant (L/mg). *K*_F_ is the constant of adsorption capacity (mg/g)(L/mg)^1/n^, and 1/*n* is the constant depicting the adsorption intensity. *R* is the universal gas constant (8.314 J/mol K), *T* is the absolute temperature (K), *b*_T_ is the constant related to the heat of adsorption (J/mol), and *A*_T_ is the equilibrium binding constant corresponding to the maximum binding energy (L/g). *q*_m_ is the theoretical saturation adsorption capacity (mg/g), *K*_DR_ is the activity coefficient related to mean adsorption energy (mol^2^/J^2^), and *ε* is the Polanyi potential (J/mol)^14^. Graphs of linear Langmuir, Freundlich, Temkin, and Dubinin-Radushkevich isotherms were plotted by *C*_e_/*q*_e_ versus *C*_e,_ log *q*_e_ versus log* C*_e_, *q*_e_ versus ln* C*_e_, and ln *q*_e_ versus *ε*^2^, respectively whereas graphs of their nonlinear were plotted by *q*_e_ versus *C*_e_^39^.

For adsorption isotherm experiments, 15 g/L of DP or 10 g/L of DPF or 7.5 g/L of CDP, or 5 g/L of CDPF were added to 500 mL Erlenmeyer flasks with initial lead concentrations from 10 to 70 mg/L. The control condition of DP or DPF or CDP or CDPF was a sample volume of 200 mL, a shaking speed of 200 rpm, pH 5, a temperature of 25 °C, and a contact time of 4 h for DP, 3 h for DPF, 3 h for CDP, and 2 h for CDPF.

### Adsorption kinetics

The adsorption kinetics of duck eggshell powder (DP), duck eggshell powder mixed iron (III) oxide-hydroxide (DPF), calcined duck eggshell powder (CDP), and calcined duck eggshell powder mixed iron (III) oxide-hydroxide (CDPF) were identified by various adsorption kinetics of linear and nonlinear pseudo-first-order kinetic, pseudo-second-order kinetic, elovich, and intraparticle diffusion models to describe their adsorption rates and mechanisms. Moreover, their adsorption kinetic equations are calculated by Eqs. (10)–(16)^40–43^:

Pseudo-first-order kinetic model:

10 $${\text{Linear}}:\;\ln \, \left( {q_{{\text{e}}} - q_{{\text{t}}} } \right) = \ln q_{{\text{e}}} - k_{1} t$$

11 $${\text{Nonlinear}}:q_{{\text{t}}} = q_{{\text{e}}} (1 - e^{{ - k_{1} t}} )$$

Pseudo-second-order kinetic model:

12 $${\text{Linear}}: \;t/q_{{\text{t}}} = 1/k_{2} q_{{\text{e}}}^{2} + \, \left( {t/q_{{\text{e}}} } \right)$$

13 $${\text{Nonlinear}}:\;q_{{\text{t}}} = k_{2} q_{{\text{e}}}^{2} t/\left( {1 + \, q_{{\text{e}}} k_{2} t} \right)$$

Elovich model:

14 $${\text{Linear}}:\;q_{{\text{t}}} = 1/\beta \ln \alpha \beta + 1/\beta \ln t$$

15 $${\text{Nonlinear}}:\;q_{t} = \beta \ln t + \beta \ln \alpha$$

Intraparticle diffusion model:

16 $${\text{Linear}}\;{\text{and}}\;{\text{nonlinear}}:\;q_{{\text{t}}} = k_{{\text{i}}} t^{0.5} + C_{{\text{i}}}$$

where *q*_e_ is the amount of adsorbed lead on adsorbent materials (mg/g), *q*_t_ is the amount of adsorbed lead at the time (*t*) (mg/g), *k*_1_ is a pseudo-first-order rate constant (min^−1^), and *k*_2_ is a pseudo-second-order rate constant (g/mg∙min). *α* is the initial adsorption rate (mg/g/min) and *β* is the extent of surface coverage (g/mg). *k*_i_ is the intraparticle diffusion rate constant (mg/g∙min^0.5^) and *C*_i_ is the constant that gives an idea about the thickness of the boundary layer (mg/g). Graphs of linear pseudo-first-order, pseudo-second-order, elovich, and intraparticle diffusion models were plotted by ln (*q*_e_ − *q*_t_) versus time (*t*), *t*/*q*_t_ versus time (*t*), *q*_t_ versus ln *t*, and *q*_t_ versus time (*t*^0.5^), respectively whereas their nonlinear graphs were plotted by the capacity of lead adsorbed by adsorbent materials at the time (*q*_t_) versus time (*t*)^39^.

For adsorption kinetic experiments, 15 g/L of DP or 10 g/L of DPF or 7.5 g/L of CDP, or 5 g/L of CDPF were added to 1000 mL of breaker with the initial lead concentration of 50 mg/L. The control condition of DP or DPF or CDP or CDPF was a sample volume of 1000 mL, a shaking speed of 200 rpm, pH 5, a temperature of 25 °C, and a contact time of 6 h.

### Desorption experiments

The desorption experiments of duck eggshell materials were studied to investigate the possible material reusability which is referred from the study of Praipipat et al.^14^. The adsorption–desorption experiments in 5 cycles were used for confirming the abilities of duck eggshell powder (DP), duck eggshell powder mixed iron (III) oxide-hydroxide (DPF), calcined duck eggshell powder (CDP), and calcined duck eggshell powder mixed iron (III) oxide-hydroxide (CDPF) for lead adsorption. The saturated DP or DPF or CDP or CDPF after lead adsorption was added to 500 mL of Erlenmeyer flask containing 200 mL of 0.5 M HNO_3_ solution, then it was shaken by an incubator shaker (New Brunswick, Innova 42, USA) at 200 rpm for 4 h. After that, it was washed with deionization water and dried at room temperature, and DP or DPF or CDP or CDPF is ready for the next adsorption cycle. Equation (17) was used for calculating the desorption efficiency in percentage.

17 $${\text{Desorption}}\;\left( \% \right) = \left( {q_{{\text{d}}} /q_{a} } \right) \times 100$$

where *q*_*d*_ is the amount of lead desorbed (mg/mL) and *q*_a_ is the amount of lead adsorbed (mg/mL).

## Result and discussion

### The physical characteristics of duck eggshell materials

**Figure 2:**
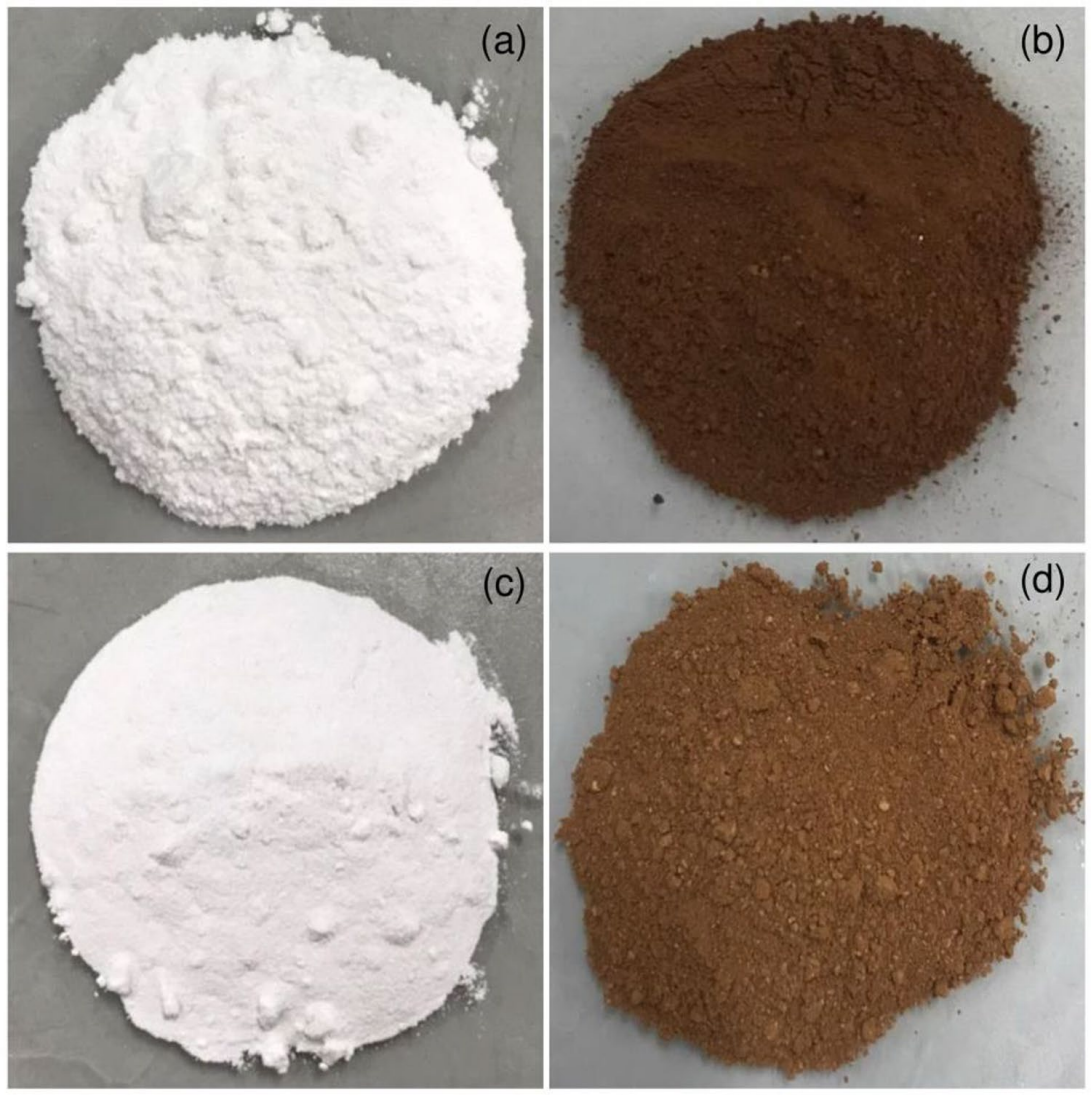
The physical characteristics of (**a**) duck eggshell powder (DP), (**b**) duck eggshell powder mixed iron (III) oxide-hydroxide (DPF), (**c**) calcined duck eggshell powder (CDP), and (**d**) calcined duck eggshell powder mixed iron (III) oxide-hydroxide (CDPF).

The physical characteristics of duck eggshell powder (DP), duck eggshell powder mixed iron (III) oxide-hydroxide (DPF), calcined duck eggshell powder (CDP), calcined duck eggshell powder mixed iron (III) oxide-hydroxide (CDPF) are demonstrated in Fig. 2a–d. DP was a white color powder demonstrated in Fig. 2a while DPF was a dark brown color powder which might be from the color of iron (III) oxide-hydroxide color added illustrated in Fig. 2b. For CDP, it was a white color similar to DP, but it was a finer powder than DP shown in Fig. 2c. Finally, CDPF was a light brown color powder shown in Fig. 2d. Therefore, a calcination process might affect to the material colors and their characteristics.

### Characterizations of duck eggshell materials

#### BET

The specific surface area, pore sizes, and pore volumes of duck eggshell powder (DP), duck eggshell powder mixed iron (III) oxide-hydroxide (DPF), calcined duck eggshell powder (CDP), and calcined duck eggshell powder mixed iron (III) oxide-hydroxide (CDPF) by BET analysis are demonstrated in Table 3. For DP and DPF, their specific surface area, pore volumes, and pore sizes were 0.955 m^2^/g, 0.0009 cm^3^/g, 3.703 nm and 12.313 m^2^/g, 0.0081 cm^3^/g, 2.617 nm which DPF had a higher specific surface area and pore volume approximately than 13-fold and ninefold of DP, whereas its pore size was smaller than approximately 1.4-fold of DP. Thus, the addition of iron (III) oxide-hydroxide helped to increase the specific area and pore volume with decreasing pore size^14,44–46^. For CDP, its specific surface area, pore volume, and pore size were 10.781 m^2^/g, 0.0065 cm^3^/g, and 2.540 nm which had the higher specific surface area and pore volume of approximately 11-fold and 7-fold of DP, and it had smaller pore size approximately 1.45-fold than DP which might result from the calcination process similar to previous studies^47,48^. For CDPF, its specific surface area, pore volume, and pore size were 34.930 m^2^/g, 0.0943 cm^3^/g, and 2.092 nm which demonstrated the highest specific surface area and pore volume with the smallest pore size than other materials resulting in the high lead adsorption capacity. Therefore, the calcination process along with adding iron (III) oxide-hydroxide is recommended to increase the specific surface area and pore volume with a small pore size for higher lead adsorption by duck eggshells than only the calcination process or adding iron (III) oxide-hydroxide. Moreover, since their pore sizes were in a range of 2–5 nm, all materials were mesoporous materials by the classification by the International Union of Pure and Applied Chemistry (IUPAC)^49^.

**Table 3:** The specific surface area, pore size, and pore volume of duck eggshell powder (DP), duck eggshell powder mixed iron (III) oxide-hydroxide (DPF), calcined duck eggshell powder (CDP), and calcined duck eggshell powder mixed iron (III) oxide-hydroxide (CDPF).

Materials	Specific surface area (m^2^/g)	Pore volume (cm^3^/g)	Pore size (nm)
DP	0.955	0.0009	3.703
DPF	12.313	0.0081	2.617
CDP	10.781	0.0065	2.540
CDPF	34.930	0.0943	2.092

#### XRD

**Figure 3:**
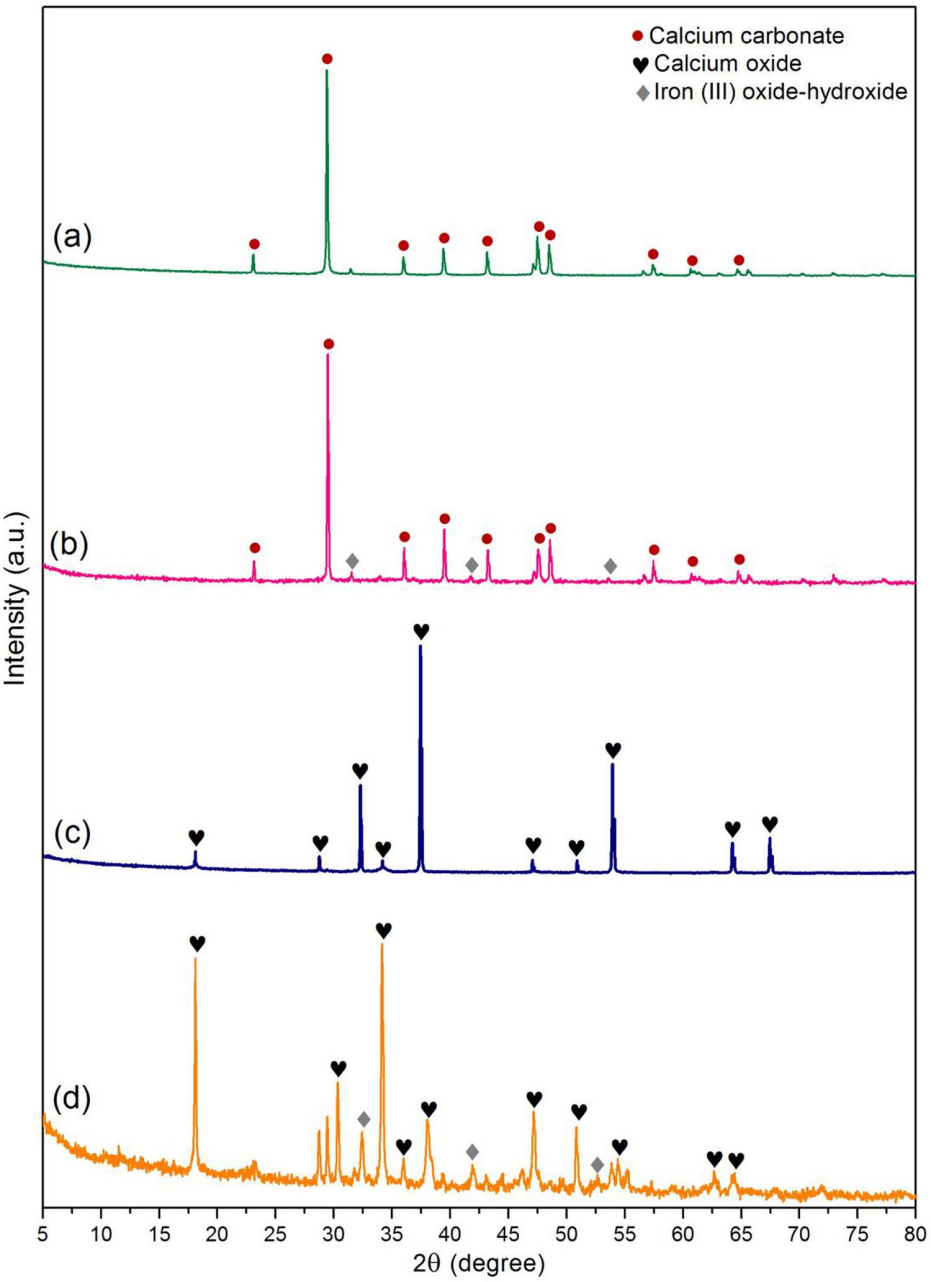
The crystalline formations of (**a**) duck eggshell powder (DP), (**b**) duck eggshell powder mixed iron (III) oxide-hydroxide (DPF), (**c**) calcined duck eggshell powder (CDP), and (**d**) calcined duck eggshell powder mixed iron (III) oxide-hydroxide (CDPF).

The crystalline structures of duck eggshell powder (DP), duck eggshell powder mixed iron (III) oxide-hydroxide (DPF), calcined duck eggshell powder (CDP), and calcined duck eggshell powder mixed iron (III) oxide-hydroxide (CDPF) by XRD analysis are presented in Fig. 3a–d. For DP and DPF, they demonstrated semi-crystalline structures with the specific calcium carbonate peaks of 23.08°, 29.40°, 35.98°, 39.41°, 43.18°, 47.50°, 48.48°, 57.40°, 60.62°, and 64.58° corresponded to JCPDS No. 05-0586^14^. For CDP and CDPF, they illustrated semi-crystalline structures by observing the specific calcium oxide peaks of 18.12°, 28.70°, 32.29°, 34.16°, 37.46°, 47.05°, 50.90°, 53.94°, 64.24°, and 67.46° matched to JCPDS No. 01-077-2010^50^. Moreover, DPF and CDPF observed the specific iron (III) oxide-hydroxide peaks of 33.92°, 41.80°, and 53.76° following JCPDS No. 29-0713^14^ confirming iron (III) oxide-hydroxide added into DPF and CDPF.

#### FESEM-FIB and EDX

**Figure 4:**
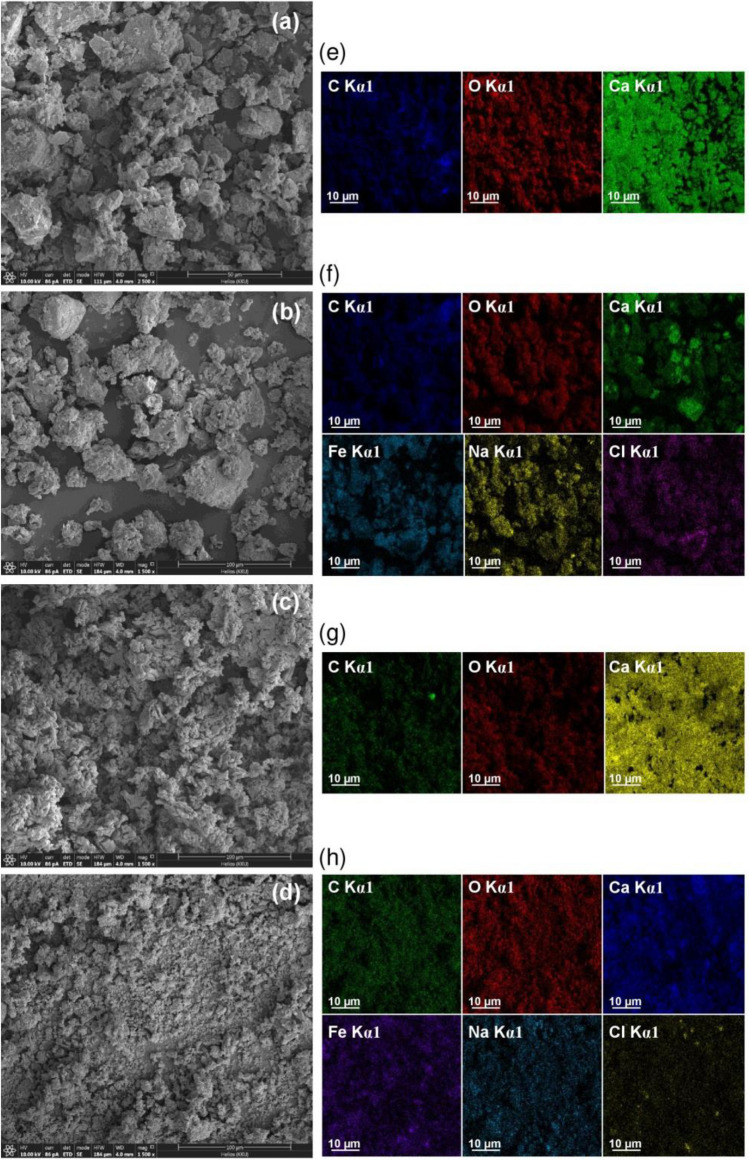
The surface morphologies and the distributions of EDX mapping of (**a**,**e**) duck eggshell powder (DP), (**b**,**f**) duck eggshell powder mixed iron (III) oxide-hydroxide (DPF), (**c**,**g**) calcined duck eggshell powder (CDP), and (**d**,**h**) calcined duck eggshell powder mixed iron (III) oxide-hydroxide (CDPF).

The surface morphologies of duck eggshell powder (DP), duck eggshell powder mixed iron (III) oxide-hydroxide (DPF), calcined duck eggshell powder (CDP), and calcined duck eggshell powder mixed iron (III) oxide-hydroxide (CDPF) by FESEM-FIB analysis at 1500× magnification with 100 µm illustrated in Fig. 4a–d. For DP and DPF, they were irregular structures with heterogeneous particle sizes, so iron (III) oxide-hydroxide added to DPF did not affect its surface morphology similar reported by another study^14^. In addition, the distributions of EDX mapping of DP and DPF are demonstrated in Fig. 4e,f. Carbon (C), oxygen (O), and calcium (Ca) were found in DP and DPF, whereas iron (Fe), sodium (Na), and chloride (Cl) were found in only DPF which might be from chemicals used in a process of the addition of iron (III) oxide-hydroxide similar to other studies^14,28,39,51^. For CDP and CDPF, their surfaces were irregular shapes similar to DP and DPF; however, they were smaller in size than DP and DPF which might result from a calcination process. The smaller particle sizes of CDP and CDPF might support higher lead adsorptions than DP and DPF similarly reports by other studies that calcined eggshells had a higher or developer porous structure than non-calcined eggshells^48^. Moreover, adding iron (III) oxide-hydroxide also did not affect the surface structure of CDPF similar to DPF. The distributions of EDX mapping of CDP and CDPF are demonstrated in Fig. 4g,h which found the same chemical elements of C, O, and Ca similar to DP and DPF, whereas CDPF had the same chemical elements as DPF with observing iron distribution on the surface of DPF and CDPF.

The chemical compositions of duck eggshell powder (DP), duck eggshell powder mixed iron (III) oxide-hydroxide (DPF), calcined duck eggshell powder (CDP), and calcined duck eggshell powder mixed iron (III) oxide-hydroxide (CDPF) by EDX analysis are reported in Table 4, and their distributions of EDX mapping are demonstrated in Fig. 4e–h. Carbon (C), oxygen (O), and calcium (Ca) were the main chemical components of all materials, whereas iron (Fe), sodium (Na), and chloride (Cl) were only found in DPF and CDPF which could be confirmed the successful addition of iron (III) oxide-hydroxide in both materials. For DP and DPF comparison, the mass percentages by weight of O, Ca, and C of DPF were decreased, whereas the mass percentages by weight of Fe, Na, and Cl were increased which might be from chemicals ferric chloride hexahydrate (FeCl_3_·6H_2_O) and sodium hydroxide (NaOH) used for the DPF synthesis. For DP and CDP comparison, the mass percentages by weight of O and C of CDP were decreased, whereas the mass percentage by weight of Ca was increased resulting from the effect of the calcination process similar to another study^52^. For CDP and CDPF comparison, the mass percentages by weight of O, Ca, and C were decreased. While, the mass percentages by weight of Fe, Na, and Cl were increased similar reason to DPF from chemicals used in the CDPF synthesis.

**Table 4:** The chemical compositions of duck eggshell powder (DP), duck eggshell powder mixed iron (III) oxide-hydroxide (DPF), calcined duck eggshell powder (CDP), and calcined duck eggshell powder mixed iron (III) oxide-hydroxide (CDPF).

Materials	O	Ca	C	Fe	Na	Cl
DP	44.1	36.4	19.5			
DPF	28.3	26.8	18.9	25.6	0.3	0.1
CDP	43.9	41.3	14.8			
CDPF	25.5	34.2	12.9	27.1	0.2	0.1

#### FT-IR

**Figure 5:**
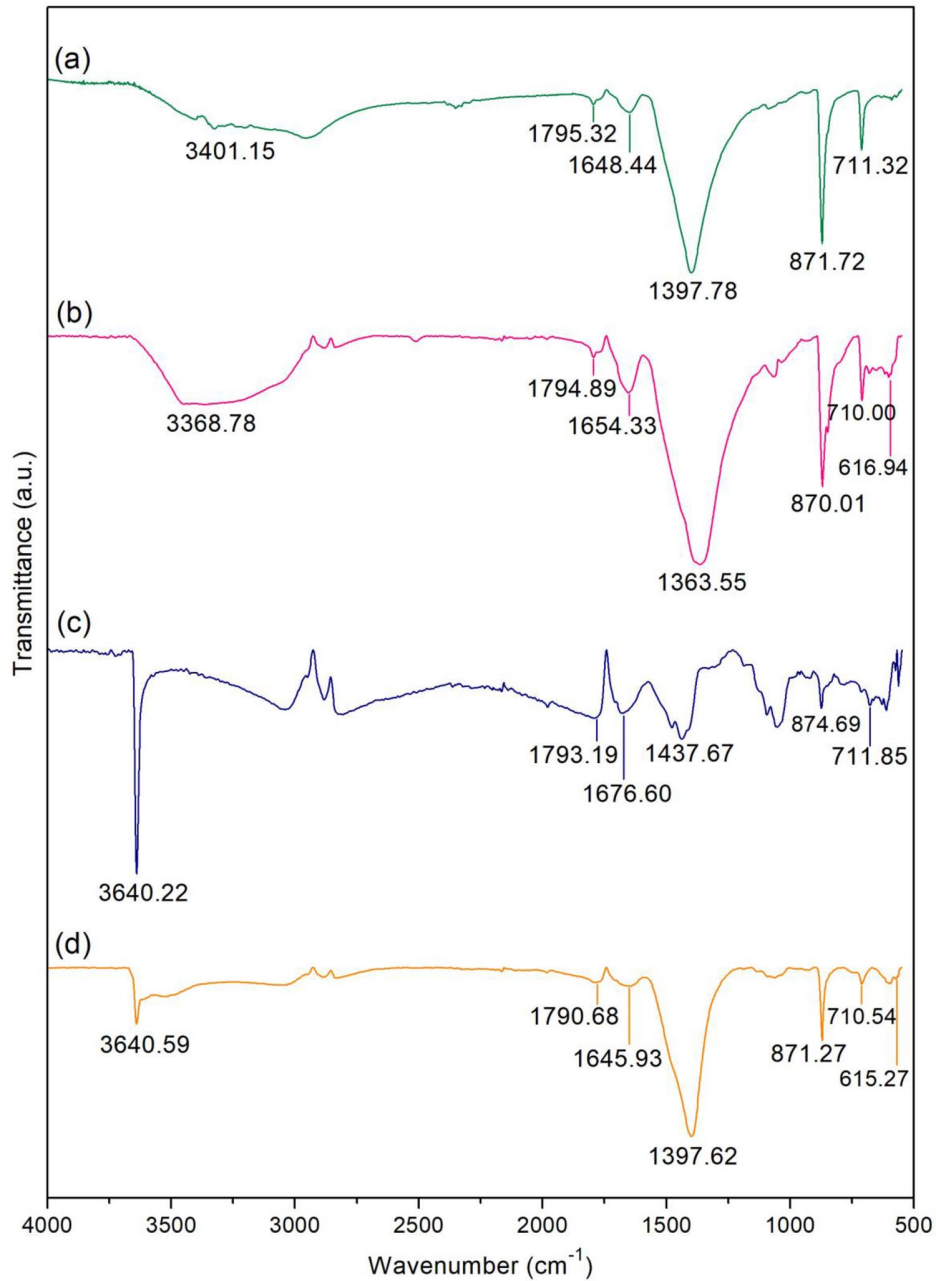
FT-IR spectra of (**a**) duck eggshell powder (DP), (**b**) duck eggshell powder mixed iron (III) oxide-hydroxide (DPF), (**c**) calcined duck eggshell powder (CDP), and (**d**) calcined duck eggshell powder mixed iron (III) oxide-hydroxide (CDPF).

FT-IR spectra of duck eggshell powder (DP), duck eggshell powder mixed iron (III) oxide-hydroxide (DPF), calcined duck eggshell powder (CDP), and calcined duck eggshell powder mixed iron (III) oxide-hydroxide (CDPF) are illustrated in Fig. 5a–d to determine their chemical functional groups. O–H, C=O, and C–O were the main chemical functional groups of all materials similar found in other studies of eggshells^48,53^, whereas Fe–O was only found in DPF and CDPF. For O–H, it was the stretching of the hydroxyl group or the water molecule^53^. For C=O, it was the stretching of the carbonate group (CO_3_^2−^) and C–O was the stretching of calcium carbonate (CaCO_3_) or bending out and in plane modes of CO_3_^2−^^50^. For DP, it observed the stretching of O–H at 3401.15 cm^−1^, stretching of C=O at 1795.32 cm^−1^ and 1648.44 cm^−1^, and stretching of C–O at 1397.78 cm^−1^, 871.72 cm^−1^, and 711.32 cm^−1^. For DPF, it detected the stretching of O–H at 3368.78 cm^−1^, stretching of C=O at 1794.89 cm^−1^ and 1654.33 cm^−1^, stretching of C–O at 1363.55 cm^−1^, 870.01 cm^−1^, and 710.00 cm^−1^, and the stretching of Fe–O at 616.94 cm^−1^. For CDP, it found the stretching of O–H at 3640.22 cm^−1^, stretching of C=O at 1793.19 cm^−1^ and 1676.60 cm^−1^, and stretching of C–O at 1437.67 cm^−1^, 874.69 cm^−1^, and 711.85 cm^−1^ which found the evidence of water adsorption by calcium oxide (CaO) resulting from the calcination process at a position of O–H similar to other studies^48,53^. For CDPF, it detected the stretching of O–H at 3640.59 cm^−1^, stretching of C=O at 1790.68 cm^−1^ and 1645.93 cm^−1^, stretching of C–O at 1397.62 cm^−1^, 871.27 cm^−1^, and 710.54 cm^−1^, and the stretching of Fe–O at 615.27 cm^−1^ which it also found the evidence of water adsorption by CaO similar to CDP.

#### The point of zero charge

**Figure 6:**
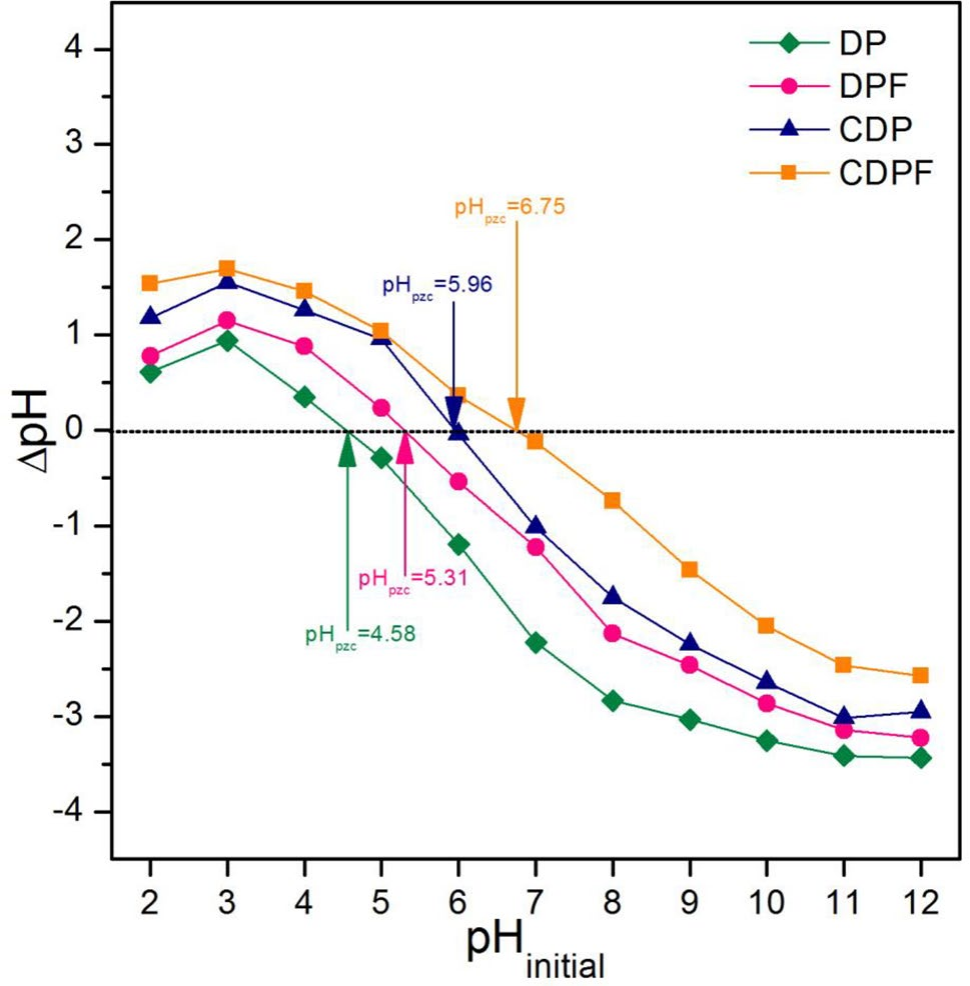
The point of zero charges of duck eggshell powder (DP), duck eggshell powder mixed iron (III) oxide-hydroxide (DPF), calcined duck eggshell powder (CDP), and calcined duck eggshell powder mixed iron (III) oxide-hydroxide (CDPF).

The point of zero charge (pH_pzc_) refers to a pH value at the net charge equal to zero of the adsorbent for realizing which pH value is good for adsorption by that adsorbent. In this study, the pH_pzc_ of duck eggshell powder (DP), duck eggshell powder mixed iron (III) oxide-hydroxide (DPF), calcined duck eggshell powder (CDP), and calcined duck eggshell powder mixed iron (III) oxide-hydroxide (CDPF) was investigated to identify which a pH value was good for lead adsorption for each material, and their results are demonstrated in Fig. 6. The pH_pzc_ values of DP, DPF, CDP, and CDPF were 4.58, 5.31, 5.96, and 6.75, respectively. As a result, the calcination process and the iron (III) oxide-hydroxide increased the pH_pzc_ of materials. Since a negatively charged material surface is preferred for capturing lead (II) ions, the pH of the solution (pH_solution_) should be higher than pH_pzc_ (pH_solution_ > pH_pzc_) to support a high lead adsorption. Therefore, the high lead adsorptions of all materials should be observed at pH > 4.

### Batch adsorption experiments

#### The effect of adsorbent dosage

**Figure 7:**
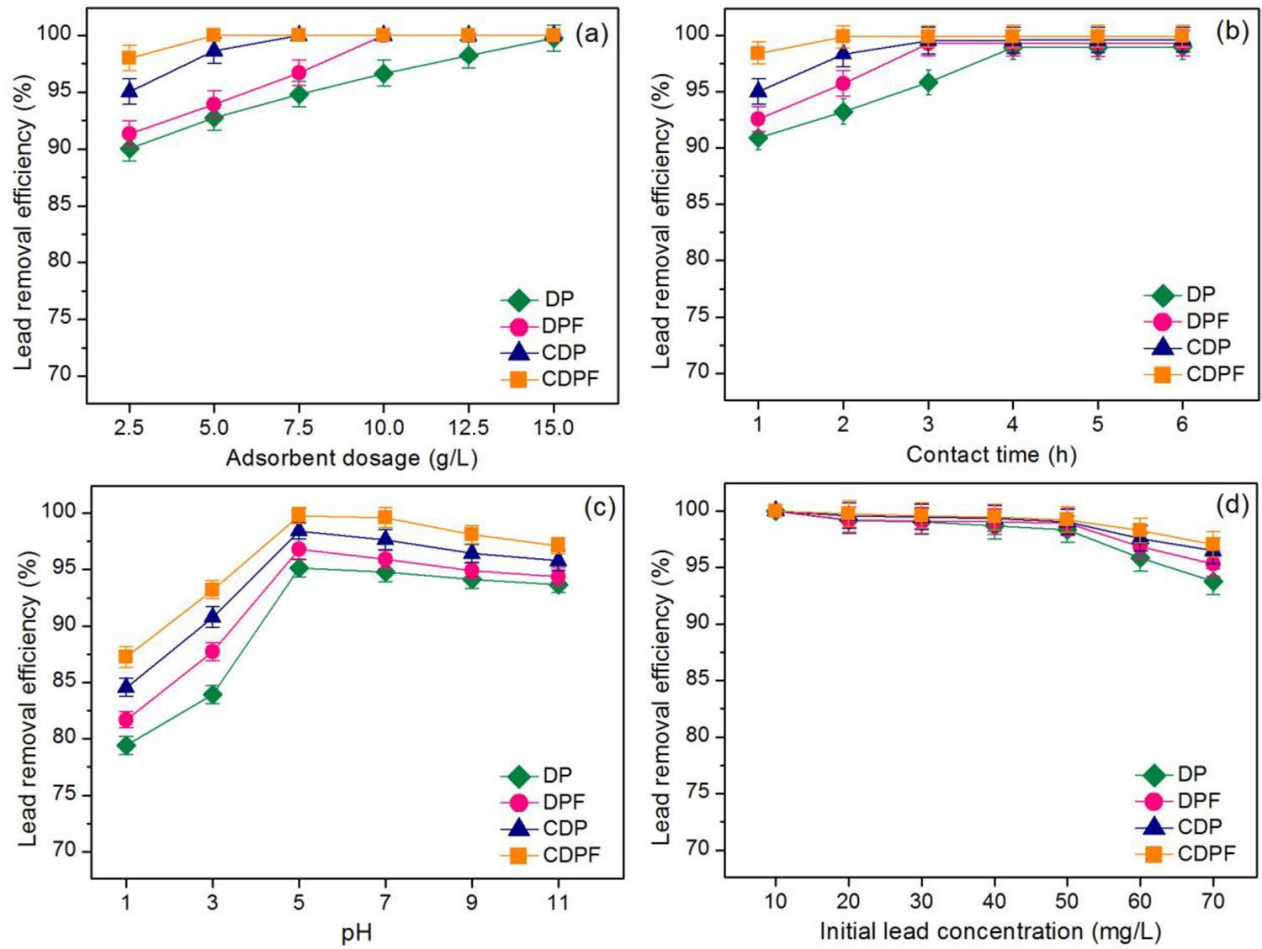
Batch experiments on the effects of (**a**) adsorbent dosage, (**b**) contact time, (**c**) pH, and (**d**) initial lead concentration of duck eggshell powder (DP), duck eggshell powder mixed iron (III) oxide-hydroxide (DPF), calcined duck eggshell powder (CDP), and calcined duck eggshell powder mixed iron (III) oxide-hydroxide (CDPF).

The dosages from 2.5 to 15 g/L of duck eggshell powder (DP), duck eggshell powder mixed iron (III) oxide-hydroxide (DPF), calcined duck eggshell powder (CDP), and calcined duck eggshell powder mixed iron (III) oxide-hydroxide (CDPF) were used for the effect of adsorbent dosage, and their results are illustrated in Fig. 7a. Their lead removal efficiencies increased with increasing of dosages which might be from increasing active sites for capturing the lead. Their highest lead removal efficiencies were 99.74% at 15 g/L for DP, 100% at 10 g/L for DPF, 100% at 7.5 g/L for CDP, and 100% at 5 g/L for CDPF, respectively. Therefore, they were used as optimum adsorbent dosages of DP, DPF, CDP, and CDPF for the effect of contact time.

#### The effect of contact time

The contact times from 1 to 6 h of duck eggshell powder (DP), duck eggshell powder mixed iron (III) oxide-hydroxide (DPF), calcined duck eggshell powder (CDP), and calcined duck eggshell powder mixed iron (III) oxide-hydroxide (CDPF) were applied for the effect of contact time, and the results are shown in Fig. 7b. Their lead removal efficiencies increased with increasing of contact time, and the highest lead removal efficiency is found at the constant contact time. Their highest lead removal efficiencies were 98.96% at 4 h for DP, 99.29% at 3 h for DPF, 99.54% at 3 h for CDP, and 99.87% at 2 h for CDPF, respectively. Therefore, they were used as the optimum contact time of DP, DPF, CDP, and CDPF for the effect of pH.

#### The effect of pH

The pH values of 1, 3, 5, 7, 9, and 11 of duck eggshell powder (DP), duck eggshell powder mixed iron (III) oxide-hydroxide (DPF), calcined duck eggshell powder (CDP), and calcined duck eggshell powder mixed iron (III) oxide-hydroxide (CDPF) were used for the effect of pH, and the results were presented in Fig. 7c. Their lead removal efficiencies were increased with the increase of pH values from 1 to 5, then they were decreased. At pH < 5, the increase of proton (H^+^) at pH 1–3 affected low lead adsorptions of all materials because of the competition of H^+^ and Pb (II) ions (Pb^2+^) agreed with the previous studies^14,16,54^. At pH > 5, their lead removal efficiencies were decreased because the hydroxide formation of lead such as PbOH^+^ (aq), Pb_2_(OH)_3_^+^(aq), Pb(OH)_2_ (aq) similarly reported by a previous study^55^ including the occurrence of lead precipitation (Pb(OH)_2_ (s)) resulted to lead removals at high pH values. The highest lead removal efficiencies of all materials were found at pH 5 for 95.12%, 96.78%, 98.41%, and 99.76% for DP, DPF, CDP, and CDPF, respectively. These results corresponded to the results of pH_pzc_ in this study and other studies that pH > 4 illustrated the highest lead removal efficiency related to pH_pzc_ of lead removals in wastewater^6,14,28,39,56^. Therefore, pH 5 was used as the optimum pH of DP, DPF, CDP, and CDPF for the effect of concentration.

#### The effect of initial lead concentration

Initial lead concentrations from 10 to 70 mg/L of duck eggshell powder (DP), duck eggshell powder mixed iron (III) oxide-hydroxide (DPF), calcined duck eggshell powder (CDP), and calcined duck eggshell powder mixed iron (III) oxide-hydroxide (CDPF) were applied for the effect of initial lead concentration, and the results are shown in Fig. 7d. Their lead removal efficiencies of duck eggshell materials were decreased with the increasing of initial lead concentration from 10 to 70 mg/L resulting from lack active sites for adsorb lead ions similarly found by other studies^6,14,28,39,45,51,56^. Lead removal efficiencies at 50 mg/L of DP, DPF, CDP, and CDPF were 98.35%, 98.94%, 99.04%, and 99.24%, respectively, and CDPF demonstrated a higher lead removal efficiency than others.

In conclusion, 15 g/L, 4 h, pH 5, 50 mg/L, 10 g/L, 3 h, pH 5, 50 mg/L, 7.5 g/L, 3 h, pH 5, 50 mg/L, and 5 g/L, 2 h, pH 5, 50 mg/L, respectively were the optimum conditions in dose, contact time, pH, and concentration of DP, DPF, CDP, and CDPF, so CDPF demonstrated the highest lead removal efficiency at high lead removal of 99.24% than other materials because it spent less material dosage and contact time than others. In addition, they could be arranged in high material efficiency to low being CDPF > CDP > DPF > DP. Therefore, adding iron (III) oxide-hydroxide along with the calcination process improved material efficiency, and CDPF was a potential material to apply in the wastewater treatment system.

### Adsorption isotherms

**Figure 8:**
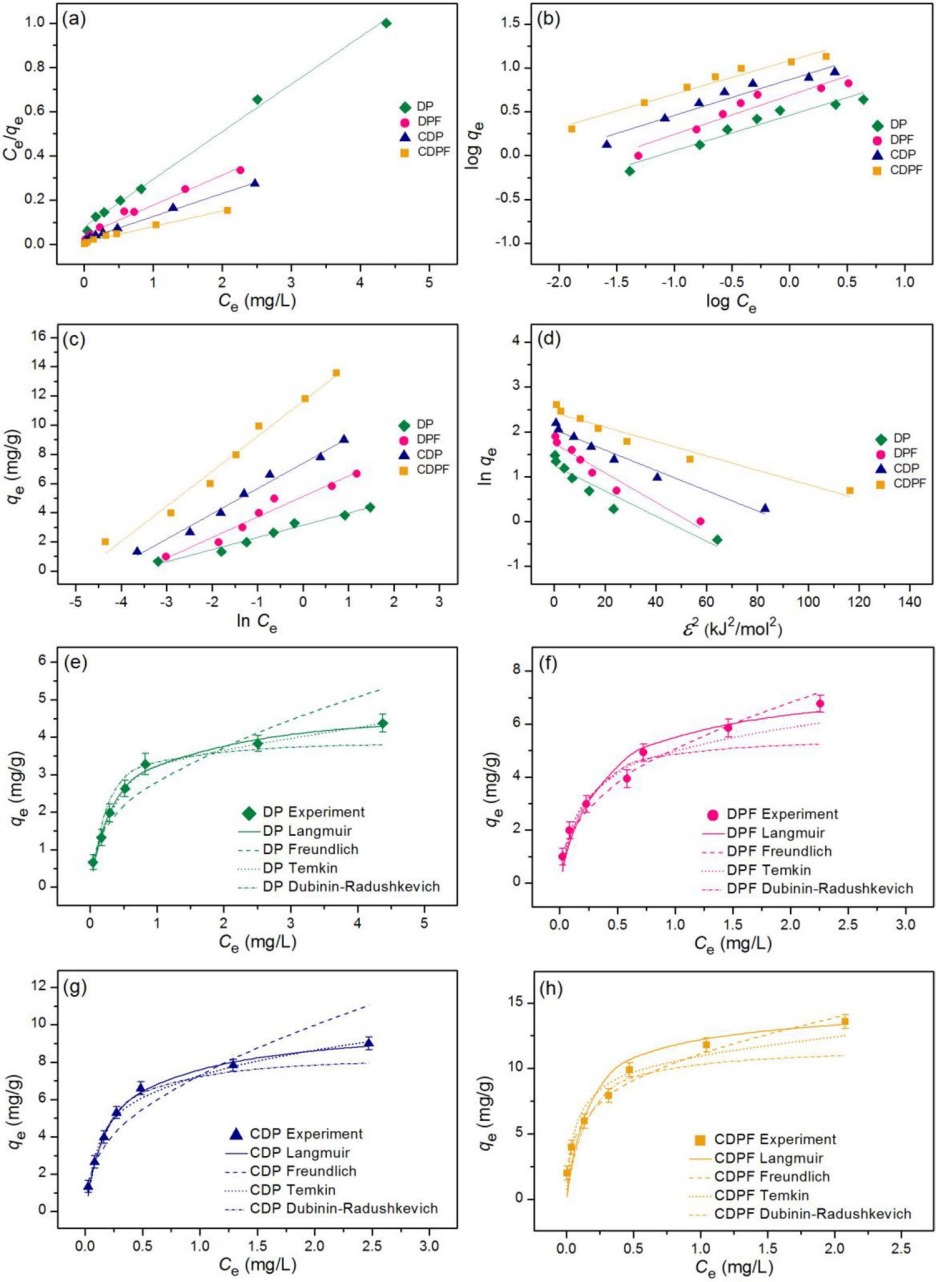
Graphs of (**a**) linear Langmuir, (**b**) linear Freundlich, (**c**) linear Temkin, (**d**) linear Dubinin–Radushkevich, and (**e**) nonlinear adsorption isotherms of duck eggshell powder (DP), (**f**) duck eggshell powder mixed iron (III) oxide-hydroxide (DPF), (**g**) calcined duck eggshell powder (CDP), and (**h**) calcined duck eggshell powder mixed iron (III) oxide-hydroxide (CDPF) for lead adsorptions.

The adsorption patterns of duck eggshell powder (DP), duck eggshell powder mixed iron (III) oxide-hydroxide (DPF), calcined duck eggshell powder (CDP), and calcined duck eggshell powder mixed iron (III) oxide-hydroxide (CDPF) for lead adsorptions were identified by linear and nonlinear models of Langmuir, Freundlich, Temkin, and Dubinin-Radushkevich models. For linear models, Langmuir, Freundlich, Temkin, and Dubinin-Radushkevich isotherms were plotted by *C*_e_/*q*_e_ versus *C*_e_, log *q*_e_ versus log *C*_e_, *q*_e_ versus ln *C*_e_, and ln *q*_e_ versus *ε*^2^, respectively. For nonlinear models, all isotherms were plotted by* C*_e_ versus *q*_e._ The plotting graph results are illustrated in Fig. 8a–h, and the equilibrium isotherm parameters are illustrated in Table 5.

**Table 5:** The comparison of linear and nonlinear isotherm parameters for lead adsorptions on duck eggshell powder (DP), duck eggshell powder mixed iron (III) oxide-hydroxide (DPF), calcined duck eggshell powder (CDP), and calcined duck eggshell powder mixed iron (III) oxide-hydroxide (CDPF).

Isotherm models	Parameters	DP	DPF	CDP	CDPF
Linear					
Langmuir	*q*_m_ (mg/g)	4.655	7.358	9.643	14.205
	*K*_L_ (L/mg)	2.672	3.259	4.548	6.579
	*R*^2^	0.996	0.977	0.998	0.988
Freundlich	1*/n*	0.405	0.410	0.417	0.422
	*K*_F_ (mg/g)(L/mg)^1/n^	2.888	5.138	7.528	11.402
	*R*^2^	0.939	0.991	0.938	0.995
Temkin	*b*_T_ (J/mol)	2974.345	1995.668	1409.381	1313.351
	*A*_T_ (L/g)	43.338	67.719	69.423	362.405
	*R*^2^	0.977	0.955	0.988	0.934
Dubinin–Radushkevich	*q*_m_ (mg/g)	3.472	5.236	7.788	10.126
	*K*_DR_ (mol^2^/J^2^)	0.028	0.027	0.023	0.019
	*E* (kJ/mol)	4.218	4.303	4.693	5.077
	*R*^2^	0.941	0.918	0.968	0.882
Nonlinear					
Langmuir	*q*_m_ (mg/g)	4.659	7.364	9.651	14.333
	*K*_L_ (L/mg)	2.688	3.259	4.562	6.723
	*R*^2^	0.998	0.978	0.998	0.989
	*R*^2^_adj_	0.997	0.974	0.997	0.986
	RMSE	0.159	0.533	0.252	1.358
Freundlich	1*/n*	0.408	0.413	0.423	0.441
	*K*_F_ (mg/g) (L/mg)^1/n^	2.901	5.145	7.534	11.436
	*R*^2^	0.942	0.992	0.943	0.996
	*R*^2^_adj_	0.930	0.990	0.932	0.995
	RMSE	0.565	0.309	1.170	1.625
Temkin	*b*_T_ (J/mol)	2987.884	2097.859	1409.381	1342.407
	*A*_T_ (L/g)	43.443	67.751	69.423	377.582
	*R*^2^	0.979	0.960	0.987	0.940
	*R*^2^_adj_	0.975	0.952	0.985	0.928
	RMSE	0.254	0.575	0.397	1.320
Dubinin–Radushkevich	*q*_m_ (mg/g)	3.827	5.364	5.292	11.216
	*K*_DR_ (mol^2^/J^2^)	0.034	0.028	0.029	0.022
	*E* (kJ/mol)	3.816	4.241	4.123	4.795
	*R*^2^	0.955	0.919	0.920	0.885
	*R*^2^_adj_	0.946	0.903	0.904	0.862
	RMSE	0.347	0.866	2.663	1.678

For linear models, the Langmuir maximum adsorption capacities (*q*_m_) of DP, DPF, CDP, and CDPF were 4.655, 7.358, 9.643, and 14.205 mg/g, and Langmuir adsorption constants (*K*_L_) of DP, DPF, CDP, and CDPF were 2.672, 3.259, 4.548, and 6.579 L/mg. For Freundlich isotherm, the 1/*n* values of DP, DPF, CDP, and CDPF were 0.405, 0.410, 0.417, and 0.422. Freundlich adsorption constants (*K*_F_) of DP, DPF, CDP, and CDPF were 2.888, 5.138, 7.528, and 11.402 (mg/g)(L/mg)^1/n^. For Temkin isotherm, *b*_T_ values of DP, DPF, CDP, and CDPF were 2974.345, 1995.668, 1409.381, and 1313.351 J/mol, and *A*_T_ values of DP, DPF, CDP, and CDPF were 43.338, 67.719, 69.423, and 362.405 L/g. For the Dubinin-Radushkevich model, the maximum adsorption capacities (*q*_m_) of DP, DPF, CDP, and CDPF were 3.472, 5.236, 7.788, and 10.126 mg/g, and the activity coefficient (*K*_DR_) values of DP, DPF, CDP, and CDPF were 0.028, 0.027, 0.023, and 0.019 mol^2^/J^2^, respectively. The adsorption energy (*E*) values of DP, DPF, CDP, and CDPF were 4.218, 4.303, 4.693, and 5.077 kJ/mol.

*R*^2^ values of DP, DPF, CDP, and CDPF on the linear Langmuir model were 0.996, 0.977, 0.998, and 0.988, respectively, and their *R*^2^ values on the linear Freundlich model were 0.939, 0.991, 0.938, and 0.995, respectively. *R*^2^ values of DP, DPF, CDP, and CDPF on the linear Temkin model were 0.977, 0.955, 0.988, and 0.934, respectively, and their *R*^2^ values on the linear Dubinin-Radushkevich model were 0.941, 0.918, 0.968, and 0.882, respectively.

For nonlinear models, the Langmuir maximum adsorption capacities (*q*_m_) of DP, DPF, CDP, and CDPF were 4.659, 7.364, 9.651, and 14.333 mg/g, and Langmuir adsorption constants (*K*_L_) of DP, DPF, CDP, and CDPF were 2.688, 3.259, 4.562, and 6.723 L/mg. For Freundlich isotherm, the 1/*n* values of DP, DPF, CDP, and CDPF were 0.408, 0.413, 0.423, and 0.441. Freundlich adsorption constants (*K*_F_) of DP, DPF, CDP, and CDPF were 2.901, 5.145, 7.534, and 11.436 (mg/g)(L/mg)^1/n^. For Temkin isotherm, *b*_T_ values of DP, DPF, CDP, and CDPF were 2987.884, 2097.859, 1409.381, and 1342.407 J/mol, and *A*_T_ values of DP, DPF, CDP, and CDPF were 43.443, 67.751, 69.423, and 377.582 L/g. For the Dubinin–Radushkevich model, the maximum adsorption capacities (*q*_m_) of DP, DPF, CDP, and CDPF were 3.827, 5.364, 5.292, and 11.216 mg/g, and the activity coefficient (*K*_DR_) values of DP, DPF, CDP, and CDPF were 0.034, 0.028, 0.029, and 0.022 mol^2^/J^2^, respectively. The adsorption energy (*E*) values of DP, DPF, CDP, and CDPF were 3.816, 4.241, 4.123, and 4.795 kJ/mol.

*R*^2^ values of DP, DPF, CDP, and CDPF on the nonlinear Langmuir model were 0.998, 0.978, 0.998, and 0.989, respectively, and their *R*^2^ values on the nonlinear Freundlich model were 0.942, 0.992, 0.943, and 0.996, respectively. *R*^2^ values of DP, DPF, CDP, and CDPF on the nonlinear Temkin model were 0.979, 0.960, 0.987, and 0.940, respectively, and their *R*^2^ values on the nonlinear Dubinin-Radushkevich model were 0.955, 0.919, 0.920, and 0.885, respectively.

*R*^2^_adj_ values of DP, DPF, CDP, and CDPF on the nonlinear Langmuir model were 0.997, 0.974, 0.997, and 0.986, respectively, and their *R*^2^_adj_ values on the nonlinear Freundlich model were 0.930, 0.990, 0.932, and 0.995, respectively. *R*^2^_adj_ values of DP, DPF, CDP, and CDPF on the nonlinear Temkin model were 0.975, 0.952, 0.985, and 0.928, respectively, and their *R*^2^_adj_ values on the nonlinear Dubinin-Radushkevich model were 0.946, 0.903, 0.904, and 0.862, respectively.

For *R*^2^ value consideration, since *R*^2^ values of DP and CDP in both linear and nonlinear Langmuir models were higher than Freundlich, Temkin, and Dubinin-Radushkevich models, its adsorption patterns corresponded to Langmuir isotherm relating to physical adsorption. While *R*^2^ values of DPF and CDPF in both linear and nonlinear Freundlich models were higher than Langmuir, Temkin, and Dubinin-Radushkevich models, their adsorption patterns corresponded to Freundlich isotherm relating to physiochemical adsorption. Since the results of linear and nonlinear of all isotherm models had close values, it recommended plotting graphs to confirm the results^34,57–63^.

Moreover, the comparison of the maximum adsorption capacity (*q*_m_) value of eggshell adsorbents for lead adsorption is illustrated in Table 6. All duck eggshell materials in this study had a higher *q*_m_ value than the studies of Alamillo-López et al.^64^, Bayu et al.^65^, Kasirajan et al.^66^, and Peigneux et al.^30^. In addition, CDPF also had a higher *q*_m_ value than the study of Hajji and Mzoughi^54^.

**Table 6:** The comparison of the maximum adsorption capacity (*q*_m_) value of eggshell adsorbents for lead adsorption.

Materials	*q*_m_ (mg/g)	References
Eggshell	3.90	^64^
Chicken eggshell	4.81	^54^
Eggshell	15.91	^67^
Eggshell	18.80	^13^
Eggshell modified with HCl	16.95	^23^
Calcined hen eggshell with silica gel	0.87	^65^
Calcined chicken eggshell	29.60	^21^
Calcined eggshell powder mixed with sericite	33.90	^20^
Calcium oxide from hen eggshell	0.88	^66^
Eggshell membranes modified with magnetic	0.07	^30^
Eggshell modified with Ag-Fe	27.80	^64^
Eggshell modified with starch and Fe_3_O_4_	57.14	^32^
DP	4.66	This study
DPF	7.36	This study
CDP	9.64	This study
CDPF	14.20	This study

### Adsorption kinetics

**Figure 9:**
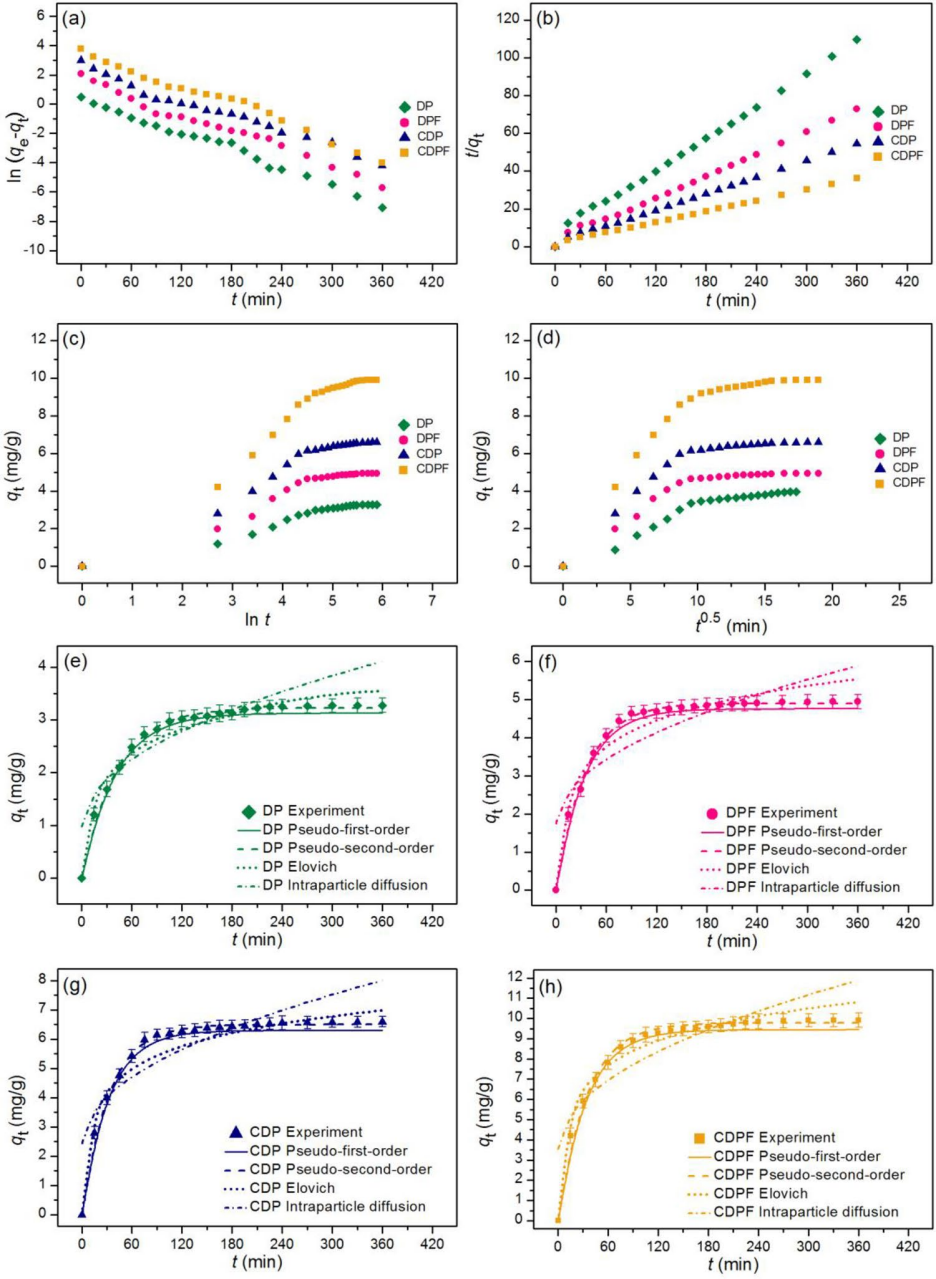
Graphs of (**a**) linear pseudo-first-order, (**b**) linear pseudo-second-order, (**c**) linear elovich model (**d**) linear intraparticle diffusion, and (**e**) nonlinear kinetic models of duck eggshell powder (DP), (**f**) duck eggshell powder mixed iron (III) oxide-hydroxide (DPF), (**g**) calcined duck eggshell powder (CDP), and (**h**) calcined duck eggshell powder mixed iron (III) oxide-hydroxide (CDPF) for lead adsorptions.

The adsorption kinetics of duck eggshell powder (DP), duck eggshell powder mixed iron (III) oxide-hydroxide (DPF), calcined duck eggshell powder (CDP), and calcined duck eggshell powder mixed iron (III) oxide-hydroxide (CDPF) for lead adsorptions were investigated to describe by linear and nonlinear kinetic models of pseudo-first-order kinetic, pseudo-second-order kinetic, elovich, and intraparticle diffusion. For linear models, they were plotted by ln (*q*_e_ − *q*_t_) versus time (*t*), *t*/*q*_t_ versus time (*t*), *q*_t_ versus ln *t*, and *q*_t_ versus time (*t*^0.5^) for pseudo-first-order kinetic, pseudo-second-order kinetic, elovich, and intraparticle diffusion models, respectively. For nonlinear models, they were plotted by *q*_t_ versus time (*t*). The plotting graph results are illustrated in Fig. 9a–h, and the adsorption kinetic parameters are presented in Table 7.

**Table 7:** The comparison of linear and nonlinear kinetic parameters for lead adsorptions on duck eggshell powder (DP), duck eggshell powder mixed iron (III) oxide-hydroxide (DPF), calcined duck eggshell powder (CDP), and calcined duck eggshell powder mixed iron (III) oxide-hydroxide (CDPF).

Kinetic models	Parameters	DP	DPF	CDP	CDPF
Linear					
Pseudo-first-order kinetic	*q*_e_ (mg/g)	2.641	3.186	3.805	8.144
	*k*_1_ (min^−1^)	0.016	0.018	0.019	0.020
	*R*^2^	0.988	0.984	0.981	0.983
Pseudo-second-order kinetic	*q*_e_ (mg/g)	3.505	5.015	6.892	10.730
	*k*_2_ (g/mg min)	0.014	0.015	0.018	0.025
	*R*^2^	0.996	0.995	0.998	0.993
Elovich	*α* (mg/g/min)	0.671	0.712	0.830	0.857
	*β* (g/mg)	1.619	0.935	0.842	0.557
	*R*^2^	0.961	0.963	0.948	0.964
Intraparticle diffusion	*k*_i_ (mg/g min^0.5^)	0.153	0.217	0.281	0.432
	*C*_i_ (mg/g)	0.963	1.737	2.431	3.455
	*R*^2^	0.799	0.725	0.721	0.756
Nonlinear					
Pseudo-first-order kinetic	*q*_e_ (mg/g)	2.850	3.310	3.954	8.217
	*k*_1_ (min^−1^)	0.017	0.020	0.021	0.022
	*R*^2^	0.984	0.985	0.983	0.982
	*R*^2^_adj_	0.983	0.984	0.982	0.981
	RMSE	0.108	0.159	0.221	0.338
Pseudo-second-order kinetic	*q*_e_ (mg/g)	3.520	5.024	6.998	10.792
	*k*_2_ (g/mg min)	0.017	0.019	0.023	0.030
	*R*^2^	0.998	0.996	0.997	0.992
	*R*^2^_adj_	0.997	0.995	0.996	0.991
	RMSE	0.215	0.343	0.409	0.230
Elovich	*α* (mg/g/min)	0.684	0.756	0.872	0.873
	*β* (g/mg)	1.644	0.989	0.854	0.634
	*R*^2^	0.963	0.967	0.950	0.961
	*R*^2^_adj_	0.961	0.965	0.948	0.959
	RMSE	4.669	0.336	2.043	0.498
Intraparticle diffusion	*k*_i_ (mg/g min^0.5^)	0.165	0.218	0.293	0.440
	*C*_i_ (mg/g)	0.978	1.747	2.443	3.551
	*R*^2^	0.795	0.729	0.722	0.758
	*R*^2^_adj_	0.783	0.715	0.707	0.745
	RMSE	0.426	0.676	0.900	1.258

For linear models, the adsorption capacities (*q*_e_) of DP, DPF, CDP, and CDPF on a pseudo-first-order kinetic model were 2.641, 3.186, 3.805, and 8.144 mg/g, and their reaction of rate constants (*k*_1_) were 0.016, 0.018, 0.019, and 0.020 min^−1^. For a pseudo-second-order kinetic model, the adsorption capacities (*q*_e_) of DP, DPF, CDP, and CDPF were 3.505, 5.015, 6.892, and 10.730 mg/g, and their reaction of rate constants (*k*_2_) were 0.014, 0.015, 0.018, and 0.025 g/mg min. For the elovich model, the initial adsorption rates (α) of DP, DPF, CDP, and CDPF were 0.671, 0.712, 0.830, and 0.857 mg/g/min, and their extents of surface coverage (*β*) were 1.619, 0.935, 0.842, and 0.557 g/mg. For the intraparticle diffusion model, the reaction of rate constants (*k*_i_) of DP, DPF, CDP, and CDPF were 0.153, 0.217, 0.281, and 0.432 mg/g min^0.5^, and their constant *C*_i_ values were 0.963, 1.737, 2.431, and 3.455 mg/g.

*R*^2^ values of DP, DPF, CDP, and CDPF on the linear pseudo-first-order were 0.988, 0.984, 0.981, and 0.983, respectively, and their *R*^2^ values on the linear pseudo-second-order kinetic models were 0.996, 0.995, 0.998, and 0.993, respectively. In addition, *R*^2^ values of DP, DPF, CDP, and CDPF on the linear elovich model were 0.961, 0.963, 0.948, and 0.964, respectively, and their *R*^2^ values on the linear intraparticle diffusion model were 0.799, 0.725, 0.721, and 0.756, respectively.

For nonlinear models, the adsorption capacities (*q*_e_) of DP, DPF, CDP, and CDPF on a pseudo-first-order kinetic model were 2.850, 3.310, 3.954, and 8.217 mg/g, and their reaction of rate constant (*k*_1_) were 0.017, 0.020, 0.021, and 0.022 min^−1^. For a pseudo-second-order kinetic model, the adsorption capacities (*q*_e_) of DP, DPF, CDP, and CDPF were 3.520, 5.024, 6.998, and 10.792 mg/g, and their reaction of rate constants (*k*_2_) were 0.017, 0.019, 0.023, and 0.030 g/mg min. For the elovich model, the initial adsorption rates (α) of DP, DPF, CDP, and CDPF were 0.684, 0.756, 0.872, and 0.873 mg/g/min, and their extents of surface coverage (*β*) were 1.644, 0.989, 0.854, and 0.634 g/mg. For the intraparticle diffusion model, the reactions of rate constant (*k*_i_) of DP, DPF, CDP, and CDPF were 0.165, 0.218, 0.293, and 0.440 mg/g min^0.5^, and their constant *C*_i_ values were 0.978, 1.747, 2.443, and 3.551 mg/g.

*R*^2^ values of DP, DPF, CDP, and CDPF on the nonlinear pseudo-first-order kinetic model were 0.984, 0.985, 0.983, and 0.982, respectively, and their *R*^2^ values on the nonlinear pseudo-second-order kinetic model were 0.998, 0.996, 0.997, and 0.992, respectively. In addition, *R*^2^ values of DP, DPF, CDP, and CDPF on the nonlinear elovich model were 0.963, 0.967, 0.950, and 0.961, respectively, and their *R*^2^ values on the nonlinear intraparticle diffusion model were 0.795, 0.729, 0.722, and 0.758, respectively.

Moreover, *R*^2^_adj_ values of DP, DPF, CDP, and CDPF in the nonlinear pseudo-first-order kinetic model were 0.983, 0.984, 0.982, and 0.981, respectively, and their *R*^2^_adj_ values in the nonlinear pseudo-second-order kinetic model were 0.997, 0.995, 0.996, and 0.991, respectively. *R*^2^_adj_ values of DP, DPF, CDP, and CDPF in the nonlinear elovich model were 0.961, 0.965, 0.948, and 0.959, respectively, and their *R*^2^_adj_ values in the nonlinear intraparticle diffusion model were 0.783, 0.715, 0.707, and 0.745, respectively.

For *R*^2^ value consideration, since *R*^2^ values of DP, DPF, CDP, and CDPF in both linear and nonlinear pseudo-second-order kinetic models were higher than the pseudo-first-order kinetic, elovich, and intraparticle diffusion models, so their adsorption rate and mechanism of both materials corresponded to a pseudo-second-order kinetic model which was chemisorption process with heterogeneous adsorption. Finally, it also recommended plotting both linear and nonlinear kinetic models for protecting against data mistranslations^34,57–63^.

### Desorption experiments

**Figure 10:**
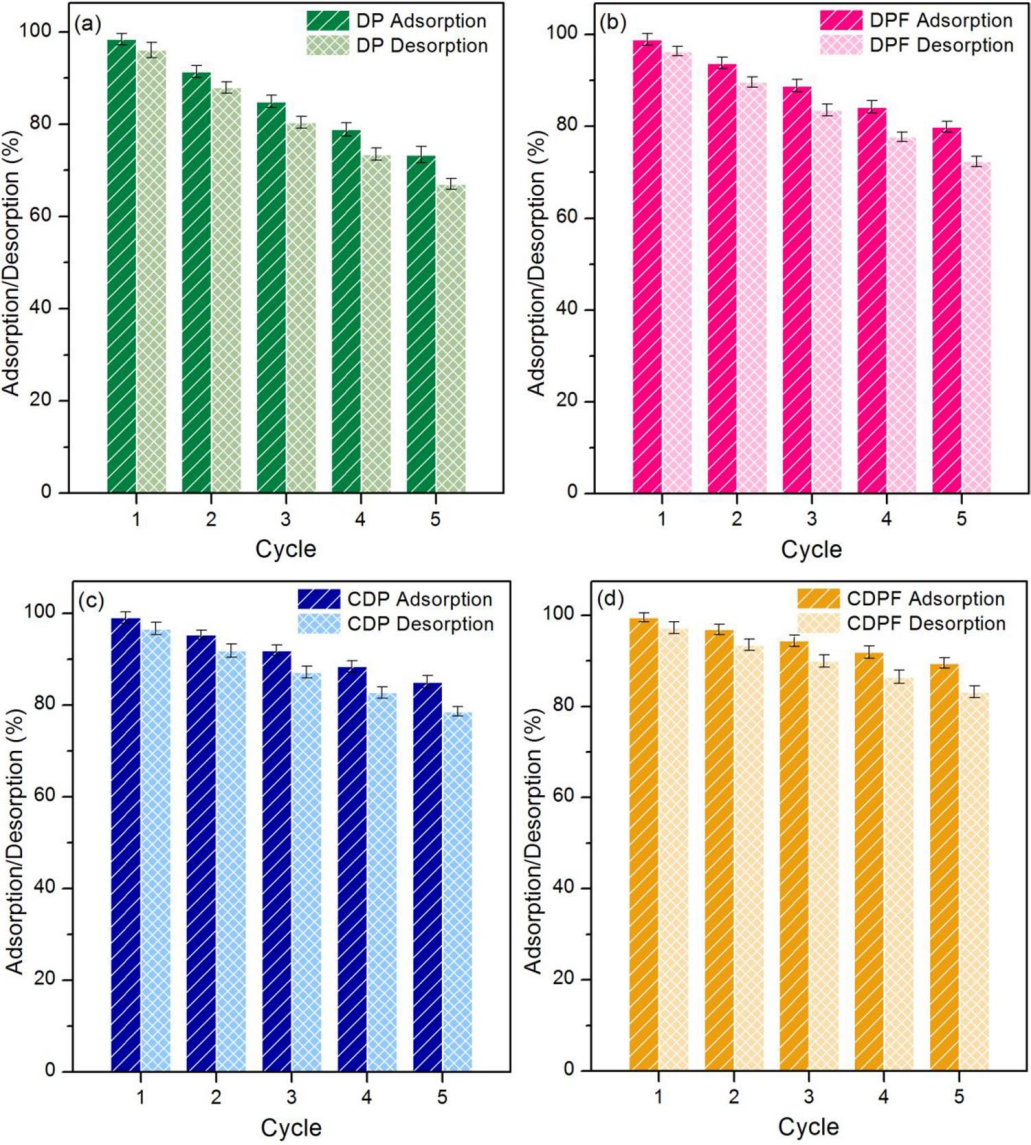
The desorption experiments of (**a**) duck eggshell powder (DP), (**b**) duck eggshell powder mixed iron (III) oxide-hydroxide (DPF), (**c**) calcined duck eggshell powder (CDP), and (**d**) calcined duck eggshell powder mixed iron (III) oxide-hydroxide (CDPF).

Before duck eggshell materials are used in industrial applications, it is necessary to estimate the cost and economics of them and whether they can be reused. As a result, the desorption experiments investigated the possible reuse of duck eggshell materials for lead adsorption. The adsorption–desorption experiments in 5 cycles were applied for this study to check the material reusability of duck eggshell powder (DP), duck eggshell powder mixed iron (III) oxide-hydroxide (DPF), calcined duck eggshell powder (CDP), and calcined duck eggshell powder mixed iron (III) oxide-hydroxide (CDPF), and their results are demonstrated in Fig. 10a–d. In Fig. 10a, DP could be reused in 5 cycles with high adsorption and desorption in ranges of 73.37–98.41% and 67.04–96.09%, respectively which adsorption and desorption were decreased by approximately 25% and 29%, respectively. For DPF, it also confirmed to be reusability in 5 cycles with high adsorption and desorption in ranges of 79.94–98.97% and 72.38–96.43%, respectively which adsorption and desorption were decreased by approximately 19% and 24%, respectively shown in Fig. 10b. For CDP could be reused in 5 cycles with high adsorption and desorption in ranges of 85.10–99.12% and 78.66–96.73%, respectively which adsorption and desorption were decreased by approximately 14% and 18%, respectively shown in Fig. 10c. For CDPF, it also confirmed to be reusability in 5 cycles with high adsorption and desorption in ranges of 89.49–99.54% and 83.19–97.23%, respectively which adsorption and desorption were decreased by approximately 10% and 14%, respectively shown in Fig. 10d. Therefore, all duck eggshell materials could be reused more than 5 cycles by more than 73%.

## The possible mechanisms of lead adsorption by duck eggshell materials

**Figure 11:**
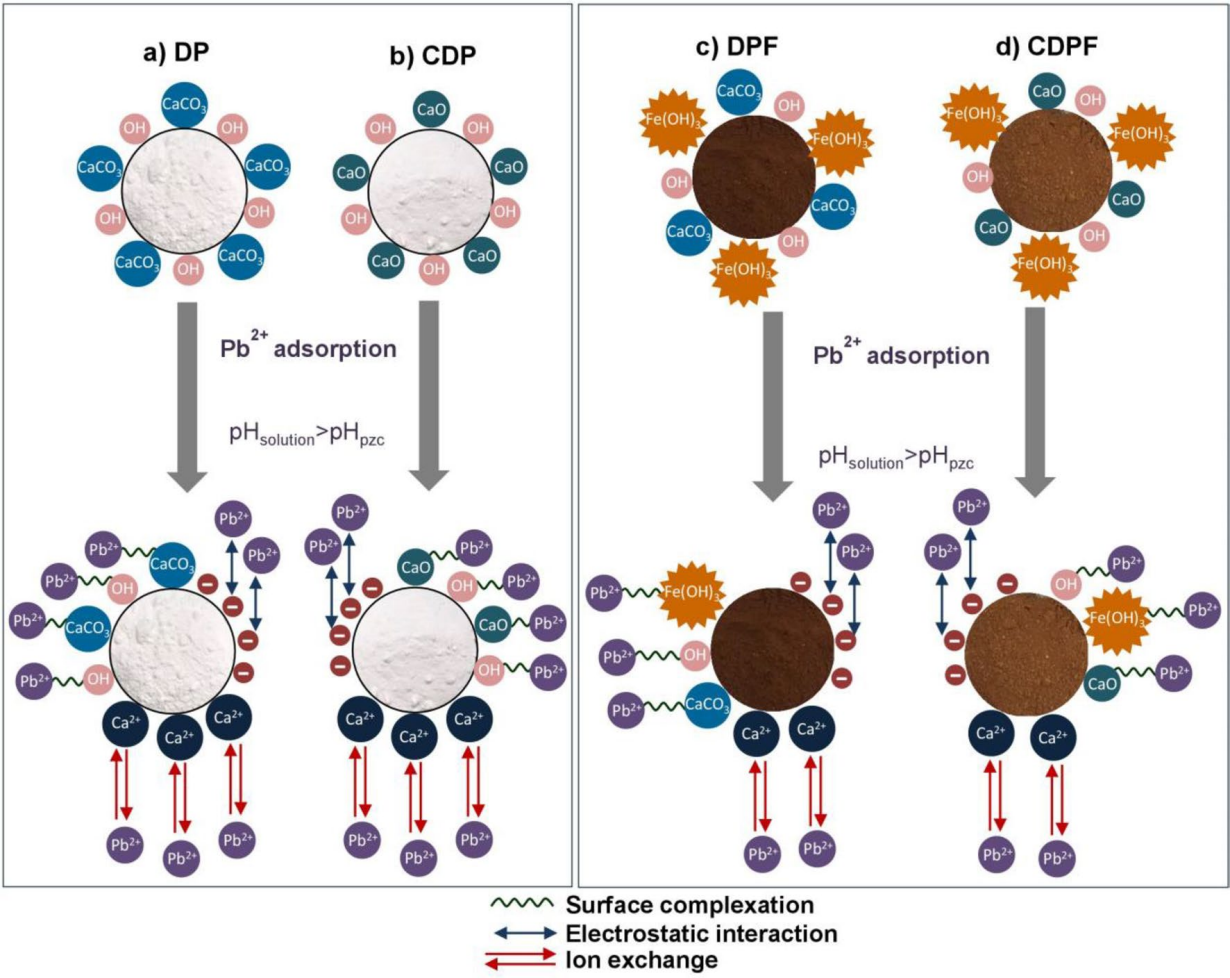
Possible mechanisms of lead adsorption on (**a**) duck eggshell powder (DP), (**b**) calcined duck eggshell powder (CDP), (**c**) duck eggshell powder mixed iron (III) oxide-hydroxide (DPF), and (**d**) calcined duck eggshell powder mixed iron (III) oxide-hydroxide (CDPF).

The possible mechanisms of lead adsorptions on duck eggshell powder (DP), duck eggshell powder mixed iron (III) oxide-hydroxide (DPF), calcined duck eggshell powder (CDP), and calcined duck eggshell powder mixed iron (III) oxide-hydroxide (CDPF) were explained by referring idea from the study of Praipipat et al.^14^ shown in Fig. 11a–d. Three main mechanisms were used for explaining their lead adsorption reactions which were surface complexation, electrostatic interaction, and ion exchange. For surface complexation, lead (II) ions (Pb^2+^) could be adsorbed by DP, DPF, CDP, and CDPF through the sharing electrons of hydroxyl ion (–OH) in calcium carbonate (CaCO_3_) or calcium oxide (CaO) on their surface and the complex compounds of DPF and CDPF from adding iron (III) oxide-hydroxide in the form of DP∙Fe(OH)_3_ or CDP∙Fe(OH)_3_. For electrostatic interaction, the surface charge of the adsorbent plays an important role in lead adsorption which depends on the pH of the solution. In addition, the point of zero charge (pH_pzc_) of the adsorbent is used to indicate which charge of the adsorbent surface is. If the pH solution is lower than pH_pzc_ (pH_solution_ < pH_pzc_), the surface charge of the adsorbent is positively charged which affects low lead adsorption because of the competition of lead (II) ions (Pb^2+^) and proton (H^+^) at an acidic pH condition. As a result, the high lead adsorption should be found at the pH of solution higher than pH_pzc_ (pH_solution_ > pH_pzc_) with a negatively charged of adsorbent surface. Since the pH_pzc_ of DP, DPF, CDP, and CDPF were 4.58, 5.31, 5.96, and 6.75, their lead adsorptions should occur at pH of solution > pH 4 by the process of electrostatic interaction^68^ agreed with the results of pH effect that the highest lead removal efficiencies of all materials were found at pH 5. For ion exchange, the substitution of calcium ions (Ca^2+^) from (CaCO_3_ or CaO) in DP, DPF, CDP, and CDPF surfaces by Pb^2+^ might happen through the ion exchange process^69^.

## Conclusion

Duck eggshell powder (DP), duck eggshell powder mixed iron (III) oxide-hydroxide (DPF), calcined duck eggshell powder (CDP), and calcined duck eggshell powder mixed iron (III) oxide-hydroxide (CDPF) were successfully synthesized. CDPF demonstrated the highest specific surface area and pore volume with the smallest pore size than other materials, so the calcination process along with adding iron (III) oxide-hydroxide helped to increase specific surface area and pore volume with decreasing pore size which supports a high lead adsorption. In addition, all materials were classified as mesoporous materials with a range pore size of 2–50 nm. DP and DPF demonstrated the semi-crystalline structures with specific calcium carbonate peaks, whereas CDP and CDPF illustrated the semi-crystalline structures with specific calcium oxide peaks. In addition, the specific iron (III) oxide-hydroxide was detected in only DPF and CDPF because of the addition of iron (III) oxide-hydroxide. Their surface morphologies were rough with irregular shapes, and the additional iron (III) oxide-hydroxide did not affect changing their surface characteristic. All materials were found carbon (C), oxygen (O), and calcium (Ca). Iron (Fe), sodium (Na), and chloride (Cl) were only found in DPF and CDPF from using chemicals in a process of addition of iron (III) oxide-hydroxide. In addition, they also found iron distribution on DPF and CDPF surfaces. They consisted of carbon (C), oxygen (O), and calcium (Ca), whereas iron (Fe), sodium (Na), and chloride (Cl) were found only in DPF and CDPF which could be confirmed the successful addition of iron (III) oxide-hydroxide in both materials. Three main function groups of O–H, C=O, and C–O were found in all materials similar found in other studies of eggshells, whereas Fe–O was only found in DPF and CDPF because of the addition of iron (III) oxide-hydroxide. The point of zero charges (pH_pzc_) of DP, DPF, CDP, and CDPF were 4.58, 5.31, 5.96, and 6.75, respectively, so the calcination process and addition of iron (III) oxide-hydroxide increased pH_pzc_ of materials. For batch experiments, the optimum conditions of DP, DPF, CDP, and CDPF were 15 g/L, 4 h, pH 5, 50 mg/L, 10 g/L, 3 h, pH 5, 50 mg/L, 7.5 g/L, 3 h, pH 5, 50 mg/L, and 5 g/L, 2 h, pH 5, 50 mg/L, respectively, and their lead removal efficiencies were 98.35%, 98.94%, 99.04%, and 99.24%, respectively. Thus, CDPF illustrated a higher lead removal efficiency than other materials because it spent less adsorbent dosage and contact time than DP, DPF, and CDP. Thus, adding iron (III) oxide-hydroxide along with the calcination process improved material efficiencies for lead adsorption. For the isotherm study, the Langmuir model was the best-fit model for DP and CDP explained by a physical adsorption process. While the Freundlich model was a good fit model for DPF and CDPF described by a physicochemical adsorption process. For the kinetic study, a pseudo-second-order kinetic model was the best-fit model for all materials related to a chemisorption process with heterogeneous adsorption. Moreover, all duck eggshell materials could reuse for more than 5 cycles for lead adsorption of more than 73%. As a result, all duck eggshell materials were high-potential materials for lead adsorption in an aqueous solution, and CDPF demonstrated the highest lead removal efficiency. Therefore, CDPF was suitable to apply for industrial wastewater treatment applications in the future.

For future works, the continuous flow study and the competing ions such as sodium (Na^+^) and magnesium (Mg^2+^) contaminated in real wastewater are recommended to study for confirming the specific lead adsorption by duck eggshell materials before applying in industrial applications.

## Data Availability

The datasets used and/or analyzed during the current study available from the corresponding author on reasonable request.
